# Extinction of threatened vertebrates will lead to idiosyncratic changes in functional diversity across the world

**DOI:** 10.1038/s41467-021-25293-0

**Published:** 2021-08-27

**Authors:** Aurele Toussaint, Sébastien Brosse, C. Guillermo Bueno, Meelis Pärtel, Riin Tamme, Carlos P. Carmona

**Affiliations:** 1 Institute of Ecology and Earth Sciences, University of Tartu, Tartu, Estonia; 2 Université Paul Sabatier, CNRS, IRD, UMR5174 EDB (Laboratoire Évolution et Diversité Biologique), Toulouse, France

**Keywords:** Biodiversity, Biogeography, Macroecology

## Abstract

Although species with larger body size and slow pace of life have a higher risk of extinction at a global scale, it is unclear whether this global trend will be consistent across biogeographic realms. Here we measure the functional diversity of terrestrial and freshwater vertebrates in the six terrestrial biogeographic realms and predict their future changes through scenarios mimicking a gradient of extinction risk of threatened species. We show vastly different effects of extinctions on functional diversity between taxonomic groups and realms, ranging from almost no decline to deep functional losses. The Indo-Malay and Palearctic realms are particularly inclined to experience a drastic loss of functional diversity reaching 29 and 31%, respectively. Birds, mammals, and reptiles regionally display a consistent functional diversity loss, while the projected losses of amphibians and freshwater fishes differ across realms. More efficient global conservation policies should consider marked regional losses of functional diversity across the world.

## Abstract (web summary)

Anthropogenic extinctions are driving functional shifts in biological communities, but these changes might differ considerably among taxa and biogeographic regions. Here the authors show that projected losses of functional diversity among land and freshwater vertebrates are unevenly distributed across the world.

## Introduction

The loss of global biodiversity is accelerating throughout the world^1^ triggering the sixth mass extinction crisis^2^. For instance, 198 extinctions of vertebrates have been recorded since 1900, which is at least ten to hundred times higher than the natural baseline rate^3^. The most recent IPBES report estimates that about 1 million of the world species (i.e. including all organisms) might be at risk of extinction in the next decades^4^. However, counts of threatened species do not fully reflect the ecological impacts of extinctions, because species’ contributions to ecosystem functioning depend on their functional traits^5,6^ (i.e. key characteristics involved in organisms’ response to the environment and their effects on the ecosystem properties^7^).

Previous mappings of the global diversity of functional traits have attempted to describe the dimensionality of the functional space of different taxa by defining a set of independent dimensions that provide non-redundant information^8–11^. For several groups of organisms, many species have evolved toward similar ecological strategies resulting in a limited set of functions permitted within the possible range of trait values. For vertebrates, functional traits can be summarized in a functional space with a few dimensions in which species are clumped around some prevalent strategies^8,11^. However, those global studies considered all the species together, whereas regions of the globe (e.g. biogeographic realms) host distinct types of ecosystems and faunas^12,13^, which possibly cover different portions of the functional space. For instance, Carmona et al.^11^ showed that among the global mammal functional spectrum, higher primate species (suborder Simiiformes) represent a functional hotspot (i.e. part of functional spectra densely occupied by species with analogous functional traits). However, the uneven and localized distribution of the higher primates might critically differentiate the regional functional space of mammals.

In the context of global changes, potential extinctions could cause dramatic erosion and rearrangement of ecological strategies globally^11^. Considering that species and their threats are not equally distributed across the world^1,14^, the functional traits of threatened species in a specific region might differ from the global pattern. As the extinction of threatened species will neither occur simultaneously across the world or at the same pace, identifying which regions are more inclined to suffer larger losses of taxonomic and/or functional diversity might help to effectively target conservation goals. Moreover, the most threatened species (classified as “CR-Critically Endangered” by the IUCN^15^) are likely to disappear earlier than species with a lower risk of extinction (“EN-Endangered”, “VU-Vulnerable” or “NT-Near Threatened” species), highlighting the need to take account the IUCN category when measuring the functional diversity at the regional scale (biogeographic realm^16^). For instance, if CR species exhibit a unique set of functional traits, their extinction would cause a drastic loss of functional diversity^11,17^. Thus, prioritizing the conservation efforts to maintain those species, might efficiently contribute to tackling the loss of biodiversity. Instead, if the most threatened species do not host a unique set of functional traits, their extinction will not drastically alter the functional diversity of species. In this case, the conservation policies aimed at conserving functional diversity should be oriented toward long-term goals, on a broader range of threatened species.

Here, we examined the distribution of the functional diversity of five groups of vertebrates across the main six terrestrial biogeographic realms by testing how regional taxonomic discrepancies translate into functional differences among realms. We then quantified how the potential extinction of species will affect the functional diversity patterns by simulating a progressive loss of threatened species from the most threatened species to the less endangered species. We characterized the functional diversity of each taxonomic group using a set of functional traits associated with different key aspects of their ecology and their life-history characteristics. The analyses reveal that the realm functional diversity differs within and between taxonomic groups. Moreover, we show that all realms are not similarly affected by the loss of threatened species but Indo-Malay and Palearctic realms are particularly inclined to experience a drastic loss of functional diversity. Understanding the regional patterns of functional diversity loss as well as the functional diversity supported by endangered species is key to improve our predictions on identifying the most vulnerable regions of the globe and hence better target the conservation policies of threatened vertebrates.

## Results

### Gathering traits, occurrences and IUCN status of vertebrates

We combined functional trait information, spatial occurrences, and IUCN status of more than 50,000 species of terrestrial mammals, birds, amphibians, reptiles, and freshwater fishes, representing 67% of the vertebrate fauna (Supplementary Table 1, Supplementary Data 1, Supplementary Fig. 1) to conduct a realm-scale analysis of current patterns of functional diversity and its erosion due to the potential loss of threatened species. We estimated the functional diversity of each realm using a probabilistic approach, providing more realistic functional spaces than other methods, such as convex-hulls^18,19^. We collected information on traits related to ecological functions (hereafter called functional traits) for the five vertebrate groups using the most recent functional trait databases^9,20–22^. In addition, we retrieved the most comprehensive available species lists compiled at the realm scale^23–25^. Because the functional trait databases for none of the groups were complete, a specific imputation procedure was used to complement this missing information (see ‘Methods’ and Supplementary Table 2). Using phylogenetic information, we imputed the values of the missing functional traits and estimated the position of the imputed species in the functional space (Supplementary Table 2). Matching species occurrences across the six biogeographic realms and functional traits showed that 67% of the species with geographic distribution information are functionally described (Supplementary Table 1). No substantial distortion toward some specific realm was identified except for reptiles in Indo-Malay where a lower proportion of species has been functionally described compared to the other realms (*χ*^2^ = 129.7, d.f. = 5, *P* < 0.001, Supplementary Table 3). The IUCN Red List (version 2020-3^15^, Supplementary Table 4) did not show strong biogeographic biases except for freshwater fishes, whose conservation status in Australian and Neotropical realms is less comprehensive than in other realms (*χ*^2^ = 1302.6, d.f. = 5, *P* < 0.001, Supplementary Table 3). In addition, the species evaluated by IUCN tend to have similar traits as non-evaluated species. Indeed, the functional overlap between all species and the subset of species evaluated by IUCN was higher than >90 % (Supplementary Table 5) suggesting that there is no marked functional bias in the IUCN Red List for most groups of species. The only exception is freshwater fishes in the Palearctic (overlap: 65%) and Neotropical (overlap: 51%) realms. These particular results should be considered with more caution.

### Current biogeographical patterns of functional diversity

**Fig. 1:**
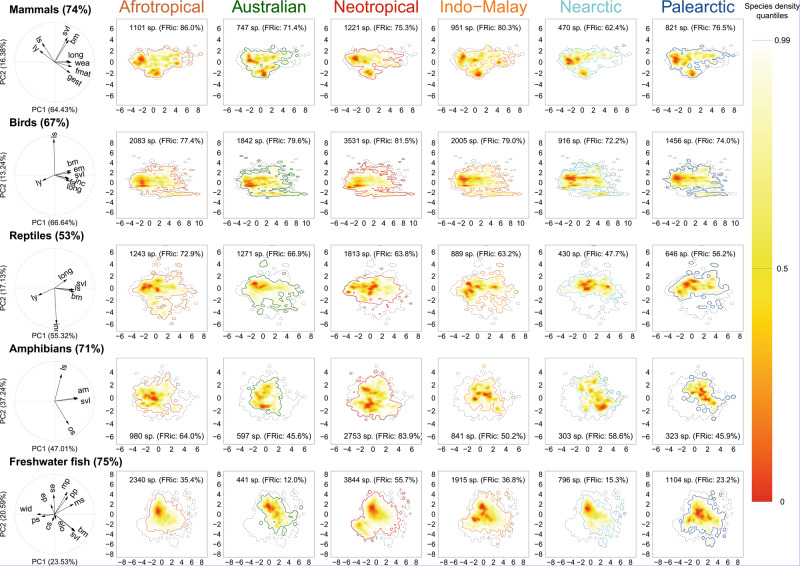
Functional spectra of vertebrates in the six biogeographic realms. Probabilistic species distributions are defined by the first two principal components of PCA considering the main functional traits for each group (see details in ‘Methods’). The correlation circles show the loadings of the considered traits in the resulting PCA for each group. Definitions of the trait abbreviations are in Table 1. For each group, the percentage of described species considered in the analysis is indicated. The colour gradient (red-yellow-white) depicts different densities of species in the functional space (i.e. red areas are more densely populated). Thick contour lines indicate the 0.99 quantiles, and thinner ones indicate quantile 0.5. The grey dotted line indicates the world’s 0.99 quantiles. The legends within each panel show the number of species and the functional richness measured as a proportion of the world’s functional richness. Global functional spectra of the six taxonomic groups (i.e. considering all species) are presented in Supplementary Fig. 3. Functional spectra and correlation circles have been made using R (codes are available online, see ‘Code availability’). Source data are provided as a Source data file.

Across all taxonomic groups, functional richness (FRic, i.e. the amount of functional space occupied by the species present in a realm) was higher in the Afrotropical, Neotropical and Indo-Malay realms and lower in the Australian, Nearctic and Palearctic realms (Fig. 1). Functional richness among realms was positively correlated to species richness for most groups (Spearman rank correlation tests, Rho >0.7, *P* < 0.001, Supplementary Table 6A) except amphibians. However, for birds, mammals and reptiles, while we observed a threefold difference in taxonomic richness among realms, functional richness was more evenly distributed. For example, 10.7% of the world’s bird species are present in the Nearctic vs. 41.2% in the Neotropical, whereas these realms contain 72.2% and 81.5% of the world’s functional richness, respectively. Mammals displayed the same pattern of evenly distributed functional richness (from 62.4% in the Nearctic to 86.0% in the Afrotropical) contrasting again with the taxonomic variability across realms (Neotropical realm had almost three times more species than the Nearctic). For reptiles, 7.6% of the world’s reptile species are present in the Nearctic but this realm hosts 47.7% of the world’s functional richness. In contrast, spatial variation of functional richness was higher for amphibians (from 45.6 to 83.9%) and freshwater fishes (from 12.0 to 55.7%), as evidenced by higher functional dissimilarities among realms (Supplementary Fig. 2).

**Fig. 2:**
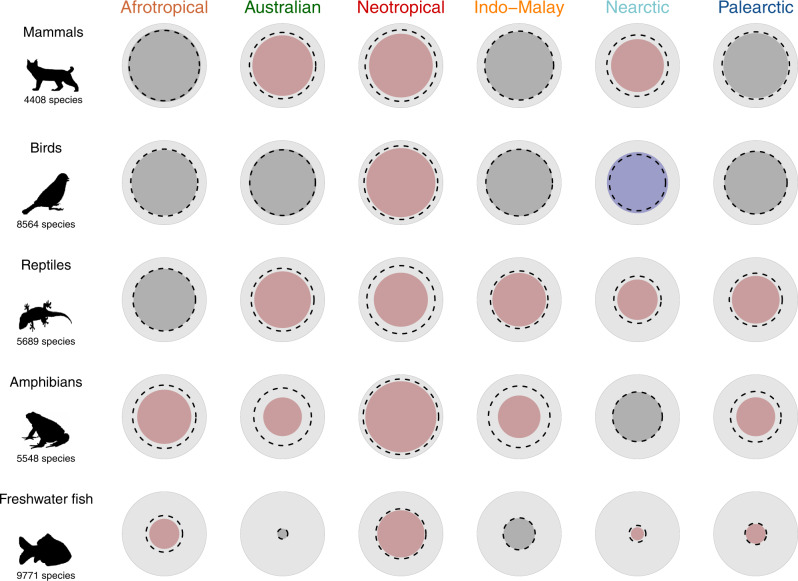
Functional richness of vertebrates in the six biogeographic realms. For each taxonomic group, the background light grey circle represents the world’s functional richness (i.e. considering all species of the group). The inner coloured circle represents the proportion of the world’s functional richness hosted by each realm. The dotted line represents the mean expected functional richness calculated with a null model where the same number of species were randomly selected from the world’s pool of species (see details in the ‘Methods’). The colour of the inner circle shows whether the functional richness was significantly higher (blue), lower (red) or not significantly different (grey) than a random expectation. Silhouettes were downloaded from PhyloPic (www.phylopic.org). Figures have been made using R (codes are available online, see ‘Code availability’). Source data are provided as a Source data file.

Since functional richness is related to taxonomic richness^26,27^, we performed null models to estimate whether the amount of functional space occupied by an assemblage within a realm is smaller (i.e. high functional redundancy) or larger (i.e. low functional redundancy) than expected given the number of species in each biogeographic realm. These results showed a generalized pattern of higher-than-expected functional redundancy (or no difference from expected), with the only exception being birds in the Nearctic (SES = 2.81, *P* < 0.001, Fig. 2, Supplementary Table 6B, Supplementary Fig. 4). This led to differences in occupancy of the functional space among realms. For reptiles, the species of the Nearctic and Palearctic mostly exhibit higher longevity and incubation times (concentrated in the upper part of the functional space; Fig. 1), while the opposite strategy was rare (at the bottom, with large portions of the functional space empty). For amphibians, the position of the functional hotspots differed between tropical and temperate realms: whereas large species are more common in temperate realms (right side of the functional space; Fig. 1), the hotspots of the tropical realms are characterized by smaller species (top left corner of the functional space; Fig. 1). For freshwater fishes, the higher functional diversity of the Neotropical realm was due to a small hotspot characterized by catfishes (family Loricariidae), present only in the Neotropics and characterized by a particular morphology and ecology (e.g. bottom mouth, adapted to browse algae^9^). For birds and mammals, three and four realms, respectively, did not differ from expected, showing that similar amounts of functional space are filled in each realm, regardless of the differences in species richness (Supplementary Fig. 5).

### Impact of loss of threatened species on functional diversity

**Fig. 3:**
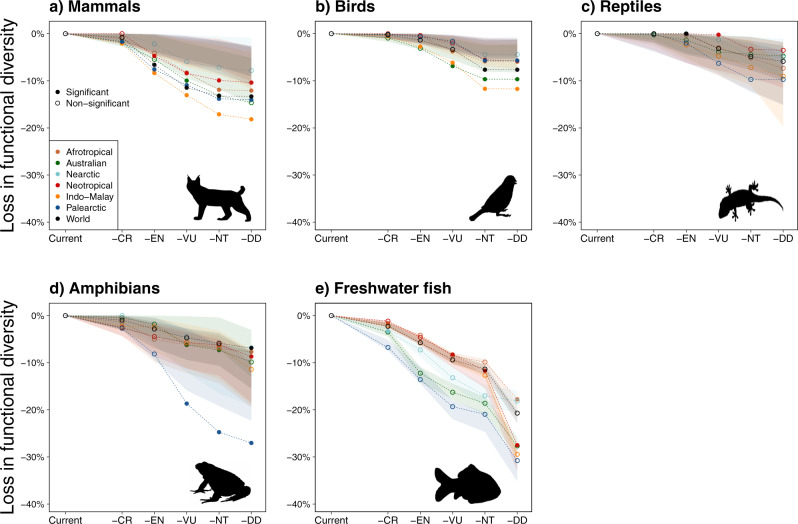
Simulated losses of functional diversity under five extinction scenarios. For each taxonomic group (panels **a**–**e**) and in each biogeographic realm (and the world), the loss of functional diversity is expressed as a percentage of the current functional diversity of the biogeographic realm (or the world). We simulated the loss of functional diversity by removing all species from the IUCN category in a progressive framework. We started removing the species with a higher risk of extinction (i.e. -CR), then we continued progressively removing the species from the categories with lower threatened risks (-EN: CR + EN, -VU: CR + EN + VU, -NT: CR + EN + VU + NT, -DD: CR + EN + VU + NT + DD). In the figure, the name of the scenario refers to the lower IUCN threat category simulated as extinct (e.g. -EN scenario considers extinctions of all CR and EN species). For each scenario, we compared the loss of functional diversity with 999 repetitions of a null model where threatened species were randomly selected among all the species present in the corresponding realm. The 999 losses of functional diversity are represented for each realm as a polygon of the confidence interval at 95%. The open circles depicted a simulated loss not significantly different than expected under the null model whereas close circles depicted a simulated loss significantly higher or lower than expected under the null model, and thus outside their correspondent polygon. Silhouettes were downloaded from PhyloPic (www.phylopic.org). Figures have been made using R (codes are available online, see ‘Code availability’). Source data are provided as a Source data file.

Simulating the loss of threatened species showed that functional diversity decreased as the number of removed species increased, but the intensity of the functional loss varied among biogeographic realms and taxonomic groups. Regardless of the extinction scenario, amphibians and freshwater fishes were the two most impacted taxonomic groups (i.e. functional loss up to 27.0% for amphibians and up to 30.8% for freshwater fishes, depending on the extinction scenarios) while reptiles were the least impacted (<10%, Fig. 3, Supplementary Fig. 6C). For mammals and birds, the Indo-Malay realm was the most strongly affected by the extinction of threatened species (functional loss up to 18.2% and 11.7%, respectively), while the Palearctic was the most affected realm for reptiles (between 0.07 and 9.7%), amphibians (between 2.7 and 27.0%), and freshwater fishes (between 6.8 and 30.8%, Fig. 3, Supplementary Fig. 6).

**Fig. 4:**
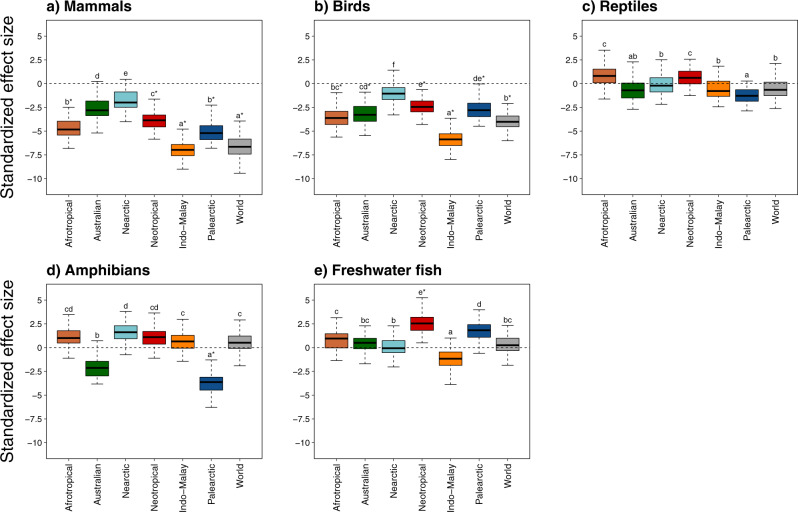
Differences in functional diversity loss among realms for the -NT extinction scenario. For each taxonomic group (panels **a**–**e**), we tested whether the standardized effect size (SES) values of the potential loss in the functional richness of each biogeographic realm significantly differ from each other using multiple pairwise comparison tests. SES distributions were obtained using a bootstrapping procedure (*n* = 1000 repetitions, see the ‘Methods’ for details). The results correspond to the -NT extinction scenario where the species considered as threatened by IUCN (i.e. CR, EN, VU and NT) were removed. Similar analyses were made for all the extinction scenarios and presented in Supplementary Fig. 9. We show for each group a compact letter displays of all pairwise comparisons with a significance level of 5%. The SES values significantly different than expected under null models are identified by an asterisk. Boxes show the median, first and third quartiles and boxes whiskers cover 95% of the distribution range. Source data are provided as a Source data file.

**Fig. 5:**
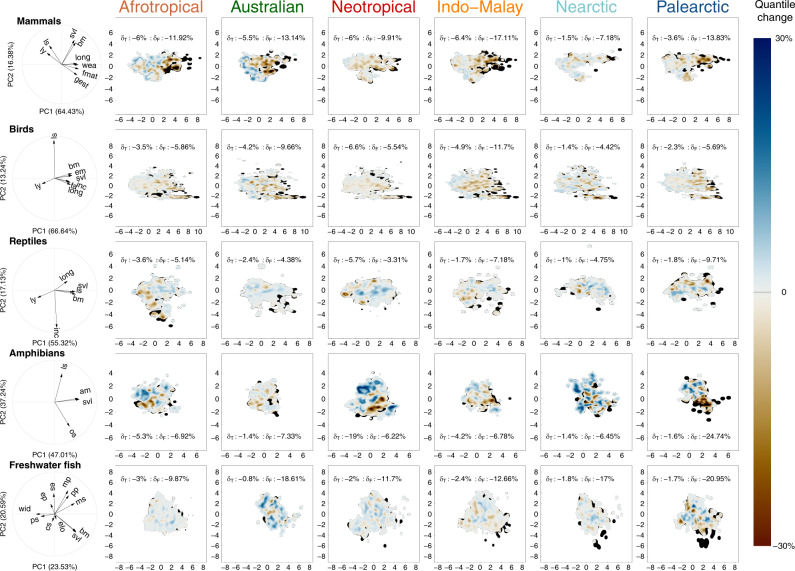
Shifts in the functional diversity after the simulated extinction of threatened species for the taxonomic groups. For each group, the shifts in functional diversity are shown for the six biogeographic realms following the -NT extinction scenario (i.e. species considered as CR, EN, VU and NT were removed). Differences (expressed in quantiles changes) are calculated between the functional spectra of species assessed by the IUCN Red List before and after removing species classified as threatened (see definition in the ‘Methods’). Brown tones reflect the threatened functional space after projected extinctions (i.e. areas representing trait values becoming relatively less frequent at the realm scale), and blue tones reflect the favourable functional space after extinctions (i.e. areas representing trait values becoming relatively more frequent at the realm scale). Black areas represent the lost functional space after extinctions. For each panel, the proportion of species (δT) and the proportion of total functional space (δF) that would be lost after extinction (expressed as a percentage of the current taxonomic and functional richness, respectively) is indicated. The functional shifts for all scenarios are in Supplementary Fig. 6 and associated values of functional diversity losses are in Supplementary Data 2. The correlation circles show the loadings of the considered traits in the resulting PCA for each group. Definitions of the trait abbreviations are in Table 1. Source data are provided as a Source data file.

We then evaluated whether the projected losses of functional richness in each realm differed from those expected for a random extinction of the same number of species from the considered realm. In other words, we tested if threatened species share particular functional traits within each realm. For that, we compared the potential loss of functional richness, considering the number of threatened species in each extinction scenario, with a simulated loss of functional richness, where the identity of the threatened species was randomized within the realm pool of species. For mammals, the potential loss of functional richness was the highest for the Indo-Malay realm, with projected losses being about two times higher than expected in all extinction scenarios except the most conservative one (Figs. 3a, 4a, Supplementary Fig. 6, Supplementary Data 2). Functional losses were concentrated toward large species with a slow pace of life in all realms, which deeply impacted the whole functional space in Afrotropical, Australian and Indo-Malay realms, by increasing the relative importance of small-sized and fast-reproducing mammals (Fig. 5). For birds, the loss of functional richness in the two most dramatic scenarios (i.e. “-NT”, and “-DD”, Fig. 3b) was higher than expected under a random loss of species (SES < 0, *P* < 0.001, Fig. 4b, Supplementary Fig. 9Db) in all realms but Nearctic. As for mammals, large and slow-living species were the most impacted, although each realm showed particular areas of the functional space that were predicted to be lost (Fig. 5). For reptiles, the loss of functional richness was not significantly different from a random loss of species in all realms even under the most dramatic scenario (Figs. 3 and 4), which means that the differences in the intensity of functional diversity loss between realms can be attributed to the number of threatened species (Fig. 3). Nevertheless, extinctions will have distinct functional consequences among realms, with a loss of large-bodied species in Indo-Malay and Australian realms, whereas extinctions will cause the loss of the small and fast-reproducing species in the Afrotropical and Neotropical realms. Moreover, in the Palearctic realm, species with both slow and fast paces of life will be at risk of extinction, therefore causing an overall loss of most extreme ecological strategies (Fig. 5). The potential loss of functional diversity in the Palearctic and Australian realms for amphibians was about four times larger than the loss observed at the global scale. It could cause deep and distinct rearrangements of the functional space among realms, with a marked loss of large and late maturity species in the Palearctic realm, whereas functional losses could be more idiosyncratic across functional space in the other realms (Fig. 5). Although not significantly different than expected (except for two realms in the most dramatic scenarios, Supplementary Fig. 9), the loss of functional diversity for freshwater fishes was high for all biogeographic realms, and comparable to the highest loss in functional diversity reported for amphibians in Palearctic. Nearctic, Indo-Malay, and Palearctic realms could lose large-bodied freshwater fish species, including predators and detritivores, whereas other realms could experience more idiosyncratic functional losses (Fig. 5).

## Discussion

We show that the functional diversity patterns of vertebrates, as well as the effect that extinctions will have on them, are unevenly distributed across biogeographic realms and taxonomic groups. The loss of species currently known to be threatened with extinction would cause a decline of up to 30% of the realms’ functional richness according to the taxonomic group and the extinction scenario considered. Some realms such as the Indo-Malay or the Palearctic are functionally more vulnerable to the loss of threatened species while other realms appear more resistant. Surprisingly, the functional vulnerability of the realms contrasted with the number of threatened species. While the Neotropical and the Afrotropical realms host a large part of the threatened species, their functional vulnerability remains limited. In contrast, the Indo-Malay realm is both taxonomically and functionally vulnerable. This demonstrates that biodiversity indices are seldom spatially congruent and should encourage conservation policies to consider together the taxonomic and functional dimensions of biodiversity to implement targeted and more effective conservation actions in the context of a global biodiversity crisis.

The loss of functional diversity among biogeographic realms can be related to the current distribution of functional diversity. Indeed, current patterns of functional space occupation underlie the evolutionary legacy of the different taxonomic groups. For mammals and birds, most of the functional spectra of the viable ecological strategies existing currently on Earth are realized in each realm. Those patterns of mammal and bird functional diversity can be related to their physiological characteristics, such as endothermy, which confer on those organisms a greater degree of independence of the environmental conditions (e.g. temperature) and allows them to extend their range across large distances. In addition, birds have the highest mobility and are least affected by geographical dispersal barriers compared to the other groups, which might explain the highest functional and taxonomical similarity between realms. For mammals, however, strategies related to large species with a slow pace of life are missing in the Palearctic and Nearctic and to a lesser extent in the Neotropical, which can be related to the decline of the megafauna during the Pleistocene^28–30^.

Despite the high functional similarity among realms for mammals, birds and reptiles, the loss of threatened species will lead to uneven functional changes among realms. Indeed, the loss of threatened species would result in a combined effect of the diversity of functions supported by the species inhabiting each realm and the number of threatened species in the same realm. For instance, the loss of threatened mammal species would have contrasting effects, with some realms (e.g. Indo-Malay) being highly impacted, with others (e.g. Nearctic) being less vulnerable. Even under a low extinction risk scenario, the potential loss of functional diversity in the Indo-Malay realm was significantly higher than the potential loss in the other biogeographic realms (Figs. 4, 5, Supplementary Fig. 6B). The causes of such loss in functional diversity for mammals are mainly linked to the erosion of the part of the functional spectrum occupied by higher primates species^11^, highly threatened in Indo-Malay, Afrotropical and Neotropical realms^31,32^ but absent from the other realms. Endangered species include our closest biological relatives in Africa, such as chimpanzees (*Pan troglodytes*), bonobos (*Pan paniscus*) and gorillas (*Gorilla* spp.), as well as orang-utans (*Pongo* spp.) in the Indo-Malay realm, and some spider (*Ateles* spp.) and capuchin monkeys (*Cebus* spp.) in the South-American tropics^31,32^. All these species are threatened by multiple anthropogenic pressures including hunting and deforestation, which might increase with ongoing and future global changes^30^. The loss of functional diversity would also be higher in the Indo-Malay realm for birds (Supplementary Figs. 6B and 9) due to erosion of the functional space occupied by large species (Fig. 5). For instance, the White-shouldered ibis (*Pseudibis davisoni*) or the Indian vulture (*Gyps indicus*) are edging very close to extinction, mainly due to habitat loss and degradation^15^. Thus, Indo-Malay does not only support a higher than expected proportion of threatened species^14^, but also a higher than expected proportion of threatened species with unique functional traits. This stresses the ultimate importance of biodiversity conservation in the Indo-Malay realm. Habitat degradation coupled to a particular geographical environment (e.g. insularity of South-East Asia) is likely to increase the threats of the large-bodied birds and mammals. As demonstrated for threatened species^14^, our results also suggest that the functional diversity of some taxonomic groups in some realms (e.g. mammals in Indo-Malay) is inherently more vulnerable to the effects of anthropogenic pressures. Despite the similar intensity of loss in functional diversity (Fig. 3), the erosion of reptiles’ functional space differed between realms (Fig. 5, Supplementary Fig. 6D). For instance, the functional diversity of the Afrotropical, Indo-Malay and Australian realms could be strongly eroded by the loss of some monitor lizards (*Varanus* spp.), one of the largest reptile species, while the loss of the vipers (*Vipera* spp.) could erode the functional space of the Palearctic. Given the importance of those large species, which are often critical consumers and keystone species^33^, for ecological processes, their extinction might affect ecosystem functioning in the near future^34,35^.

The current occupation of the functional space for amphibians and freshwater fishes is more uneven among biogeographic realms—each realm hosts a low proportion of the world’s functional diversity. This is particularly striking for amphibians and freshwater fishes where the realms’ functional spectra largely differ from the global patterns. This might reflect a functional adaptation to local environmental conditions. Although our data do not allow disentangling the processes behind such distinctiveness, we can hypothesize that the strong link of amphibians and freshwater fishes to the aquatic environment has limited their dispersal. Accordingly, amphibians and freshwater fishes have smaller distribution ranges^36^ and lower long-distance dispersal abilities than endothermal organisms like birds or mammals. For instance, the recolonization of Central and Western Europe by fish species from Ponto-Caspian Europe after the Last Glacial Maximum^37^ could explain the low functional diversity of the Palearctic realm for freshwater fish fauna. In contrast, fewer climatic restrictions and the higher rates of speciation reported in tropical realms across evolutionary times could have contributed to the exploration of unique parts of the functional space that are not realized in northern realms. For instance, Neotropical Siluriformes have unique ecological strategies, ranging from algae browsing Loricaridae (a strategy also adopted by other fish orders elsewhere, but only represented by a few species out of the Neotropics) to parasitic Trichmycteridae catfishes^9^.

For reptiles, amphibians and freshwater fishes, the changes of functional diversity among realms were stronger in both intensity and direction than those reported at the global scale^11^. For instance, many large-bodied freshwater fishes, such as sturgeons (*Acipenser* spp.), are threatened in the Palearctic, whereas many small-bodied species, such as suckermouth armoured catfishes (*Chaetostoma* spp.), are threatened in the Neotropics^15,25^. Moreover, the high current functional distinctiveness between biogeographic realms also explained the differences in the loss in functional diversity among realms. Indeed, some threatened species host a unique set of functional traits in some realms, while being functionally redundant with non-threatened species in other realms. For example, the functional traits, such as viviparity and low fecundity of some threatened amphibian species such as salamanders (*Salamandra lanzai* or *S. algira*) are unique in the Palearctic, the realm that could experience the strongest loss in functional diversity, whereas similar traits are filled by another amphibian species in tropics, such as the caecilians in Afrotropical, Neotropical and Indo-Malay^38^. Although these results were hardly affected by a potential bias due to different sampling efforts among realms and/or among functional traits, some results should be considered with caution. For example, only 21% of the freshwater fishes in Neotropical have been evaluated by IUCN versus 44% worldwide. More studies are thus required to better evaluate the influence of the IUCN Red List completion on the predicted losses of functional diversity^9,39^ for specific taxonomic groups in some incompletely evaluated realm faunas.

While large-sized species with a slow pace of life are more likely to be threatened globally^11^, discrepancies arise at the realm scale, with threatened species supporting distinct parts of the functional space in different realms. Indeed, the loss of some small-sized threatened species such as geckos (*Sphaerodactylus* spp.) in the Neotropical realm or moss frogs (*Arthroleptella* spp.) in the Afrotropical realm would contribute to the erosion of the functional richness of those two realms (Fig. 5). Such a pattern is not noticeable on a global scale because of the functional redundancy between species from different realms^11^. For instance, while most of the threatened amphibians in the Palearctic are large-sized species, making associated functional traits highly vulnerable to extinction (black areas, Fig. 5, Supplementary Fig. 6E), species with similar traits are not endangered in other realms, so that the corresponding part of the functional space does not appear as threatened using a global scale approach^11^. Further studies should focus on smaller spatial scales since we can expect that such discrepancies between global and realm scales might also occur when downgrading the spatial scale. In particular, observations at the country level would be particularly helpful to enhance the effectiveness of conservation policies, since this is the scale at which such policies are most often implemented^4^.

Even if it is not possible to predict the ecological consequences of functional loss at the realm scale, the loss of functional richness might trigger a loss of stability in the ecosystems and weaken their capacity of resistance and resilience to ongoing global changes. For instance, large-sized fish species are recognized as key species controlling food webs through predation^40^, or nutrient cycling^41^. Their local extinction in controlled experiments caused eutrophication and a drastic increase in organic matter deposition, thus degrading water quality and purification capacity^40,41^. Most of the large detritivore and carnivore fish species are under threat in the Palearctic (e.g. sturgeons and pikes), Nearctic, and Indo-Malay realms;^42^ their loss might reduce the services they provide to humanity. Although non-native species introductions might partly compensate for those functional losses, the detrimental and unpredictable effect of biological invasions^43^ makes this a high-risk strategy. The Indo-Malay realm currently experiences multiple threats including increasing habitat degradation through deforestation and damming^44,45^, as well as a strong overharvest of both terrestrial and aquatic vertebrate faunas^46–48^. Such a steep increase of environmental disturbances combined with the functional vulnerability of the Indo-Malay realm for all groups of vertebrates should elicit coordinated and efficient conservation strategies in this region. This is further exacerbated in the Indo-Malay realm due to the strong dependency on wild animal resources for food supply^49^.

Toward a global scale conservation perspective, the global loss of species should be tackled differently according to the organisms. Mammals and birds occupy similar parts of their functional space in all biogeographic realms and display similar responses to the projected species extinction. This allows a global functional approach to alleviate the predicted biodiversity loss for these two taxonomic groups. In contrast, reptiles, amphibians and freshwater fishes are distributed in different parts of their functional space in each realm, likely a result of evolution within smaller units, higher dependence on environmental conditions and/or low dispersal capacities. For these types of organisms, the projected loss of functional diversity differs from global assessment and requires more regional or local approaches to better manage the functional consequences of species extinctions.

## Methods

### Spatial database

We collected species occurrences from the most accurate and available source of data for each taxonomic group. For mammals, birds, reptiles and amphibians, we used the IUCN spatial database to assign realm identity for each species^15^. By doing this, we assigned a realm for 5489 mammal species, 10,787 bird species, 5489 reptile species and 5833 amphibian species. Since IUCN spatial database does not cover all species, we completed our database with two additional sources of species occurrences: (1) the WWF WildFinder species database^23^, except for mammals where we used the latest version of the species distribution provided by ref. ^24^. If (1) was not available, we used (2) the global biodiversity information facility (GBIF). Using WWF WildFinder, we assigned a realm for 1634 bird species, 7378 reptile species and 2006 amphibian species. 437 mammal species were assigned using ref. ^24^. From GBIF, we downloaded all the records belonging to the four classes of animals (Mammals^50^, Aves^51^, Reptiles^52^ and Amphibians^53^). Before using the spatial data, we cleaned the dataset following a cleaning procedure that was similar to but more conservative than other currently available methods (e.g. CoordinatesCleaner, BDCleaner^54^). First, records were screened, and only those with (1) coordinates; (2) a taxonomic rank of “species” were kept. From this list, we filtered out the records with clearly false locality coordinates (e.g. latitude equal to longitude, both latitude and longitude equal to 0, and longitude/latitude outside the possible range (i.e. −180; 180 for longitude and −90; 90 for latitude)). Those are the most common errors encountered with GBIF occurrence data^55^. In addition, we removed the records from living specimens (i.e. from zoos, botanical gardens), conserved specimens (i.e. museums), and unknown sources. We also excluded the species with less than 50 records within each realm as a low number of records can be due to misidentifications, which might have strong effects on our analyses. We finally refined the dataset by overlaying the occurrences within the six biogeographic realms (see below) and dropping the species that fall outside of the polygons. This spatial overlay process was conducted using the ‘sp’ library^56^ in R. The number of species for which realm was assigned using GBIF was 1 (<0.02%) for mammals, 442 (3.4%) for birds, 572 (5.3%) for reptiles and 25 (3.2%) for amphibians.

We performed additional analyses to evaluate how each database (i.e. IUCN, WWF and GBIF) influenced the functional space of each group in each realm and the world (see “World and realm functional space” below). For that, we calculated the functional overlap between the functional spaces built with all species and the functional space built with all species except the species retrieved from the IUCN spatial data (Supplementary Fig. 7A), or the species retrieved from WWF (Supplementary Fig. 7B), or the species retrieved from GBIF (Supplementary Fig. 7C). The higher the functional overlap, the less affected was the functional space by the data source (IUCN, WWF or GBIF). The functional overlap between the functional spaces built with all species and functional space built with all but the species retrieved from WWF (or from ref. ^24^ for mammals) was higher than 95% at a global and realm scales for all groups (Supplementary Fig. 7B). The functional overlap between the functional spaces built with all species and functional space built with all but the species retrieved from GBIF was higher than 99% at a global and realm scales for all groups (Supplementary Fig. 7C). This testified that the functional spaces of the realms were already well covered by the species retrieved from the IUCN spatial data.

For freshwater fishes, we collected the occurrences database from ref. ^25^, which provides the spatial distribution of about 13,000 species (out of the 17,000 described) and currently represents the most detailed spatial information on this taxa.

We considered six biogeographic realms for terrestrial and aquatic animals^16,23,25,57,58^, the Afrotropical, Australian (combining Australasian and Oceania), Nearctic, Neotropical, Indo-Malay and Palearctic (Supplementary Fig. 8 and ref. ^58^). Notice that the Australasian and Oceania realms are considered as one combined realm in our analyses since the number of records was too low in Oceania for some taxonomic groups (e.g. fishes) to consider it as an independent realm. We thus called it the Australian realm, following the terminology of ref. ^57^. Our final database of spatial occurrences for the six realms encompassed 5927 mammals, 12,863 birds, 13,439 reptiles, 7864 amphibians and 13,008 freshwater fishes occurring in at least one biogeographic realm (Supplementary Table 7).

### Functional traits

We collected information on traits related to ecological functions for the five groups of vertebrates. All the traits have been selected for their ecological relevance and gathered from published studies (see Table 1 and Methods in ref. ^11^ for details on the estimation of functional traits) and mostly related to life-history characteristics. For mammals, birds and reptiles, we used the AMIOTE database^20^ including data for 4953 species of mammals, 9802 species of birds, and 6567 species of reptiles. For mammals, we selected a subset of eight traits for which at least 1000 species were informed^11^. These traits were: litter size (number of offspring per litter), number of litters per year, adult body mass (g), longevity (years), gestation length (days), weaning length (d), time to reach female maturity (days), and distance from the tip of the snout to the tail base (cm). For birds, we selected a total of eight traits: clutch size (number of eggs), number of clutches per year, adult body mass (g), incubation time (days), longevity (years), fledging age (days), egg mass (g) and distance from the tip of the beak to the opening of the cloaca (cm). For reptiles, we selected a total of six traits with sufficient information: clutch size (number of eggs), number of clutches per year, adult body mass (g), incubation time (days), longevity (years) and distance from the tip of the snout to the opening of the cloaca (cm). For amphibians, we used the AmphiBIO database^21^ to get data for 6776 species of amphibians. Within this dataset, we selected five traits with enough information: age at maturity (years), the maximum number of reproduction events per year, body size (mm), maximum litter size (number of individuals) and offspring size (mm). Finally, for freshwater fishes, we used the last updated version of the most comprehensive database on morphological traits, available for 10,705 species of freshwater fishes^9,22^. The link between morphological traits and ecological functions is well documented for freshwater fishes^59–61^, making morphological traits a relevant proxy to describe the functional diversity of this group. This database encompasses 11 traits describing the size and shape of body parts involved in food acquisition and locomotion. The fish body shape and weight were described through the size using the standard length (cm) and body mass (g) taken directly from FishBase^62^, body elongation (ratio between body length and body depth) and body lateral shape (ratio between the head depth and body depth). The other traits describing the position and the size of each part of the fish were eye size and position, mouth size and position, pectoral fin size and position, and caudal peduncle throttling and were measured on fish pictures^9,60^.

**Table 1:** Functional traits considered in each group.

Group	Trait	Number of species (% completeness)
Mammals	ls: litter size (number of offspring per litter)	3511 (70.89%)
	ly: number of litters per year	2146 (43.44%)
	bm: adult body mass (g)	4651 (93.90%)
	long: longevity (years)	2614 (52.78%)
	gest: gestation length (days)	2220 (44.82%)
	wea: weaning length (d)	2043 (41.25%)
	fmat: time to reach female maturity (days)	2000 (40.38%)
	svl: distance from the tip of the snout to the tail base (cm).	3921 (79.16%)
Birds	ls: clutch size (number of eggs)	6892 (70.31%)
	ly: number of clutches per year	1784 (18.20%)
	bm: adult body mass (g)	9532 (97.25%)
	inc: incubation time (days)	2269 (23.15%)
	long: longevity (years)	1672 (17.06%)
	fa: fledging age (days)	1844 (18.81%)
	em: egg mass (g)	4888 (49.87%)
	svl: distance from the tip of the beak to the opening of the cloaca (cm)	1615 (16.48%)
Reptiles	ls: clutch size (number of eggs)	2675 (40.73%)
	ly: number of clutches per year	1018 (15.50%)
	bm: adult body mass (g)	2494 (37.98%)
	inc: incubation time (days)	1369 (20.85%)
	long: longevity (years)	1214 (18.49%)
	svl: distance from the tip of the snout to the opening of the cloaca (cm)	5140 (78.27%)
Amphibians	am: age at maturity (years)	384 (5.67%)
	svl: body size (mm)	5227 (77.14%)
	ls: maximum litter size (number of individuals)	1623 (23.95%)
	os: offspring size (mm)	1330 (19.63%)
Freshwater fish	es: eye size (ratio of the diameter of the eye to the head depth)	8033 (75.68%)
	ep: eye position (ratio of the centre of the eye to the bottom of the body to the body depth)	8033 (75.68%)
	ms: mouth size (ratio of the length from snout to the corner of the mouth to the head depth)	7264 (68.44%)
	mp: mouth position (ratio of the distance from the top of the mouth to the bottom of the body to the body depth)	8029 (75.65%)
	elo: body elongation (ratio of the maximum body length to the maximum body depth)	8063 (75.97%)
	wid: body lateral shape (ratio of the head depth at the vertical of the eye to the maximum body depth)	8057 (75.91%)
	ps: pectoral fin size (ratio of the length of the longest ray of the pectoral fin to body length)	7448 (70.17%)
	pp: pectoral fin position (ratio of the distance between the upper insertion of the pectoral fin to the bottom of the body to body depth)	7999 (75.36%)
	cs: caudal shape (ratio of the maximum depth of the caudal fin to the minimum depth of the caudal peduncle)	7618 (71.77%)
	svl: distance from the tip of the snout to the posterior end of the last vertebra or to the posterior end of the midlateral portion of the hypural plate (cm)	5185 (48.85%)
	bm: adult body mass (g)	1281 (11.97%)

Adapted from Carmona et al.^11^.

The completeness corresponds to the number of species for which the trait values are available, and the percentage shows the proportion of functionally informed species compared to spatially informed species.

Since none of the trait databases assembled was complete, we completed this information by performing trait-imputation procedures generated using the missForest R package^63^. Evolutionary relationships were also considered in the imputation process by including the first ten phylogenetic eigenvectors (see details in ref. ^11^). We obtained published phylogenies for each of the taxonomic groups^64–68^. Species that were not present in the phylogeny were added to the root of their genus, using the ‘add.species.to.genus’ from the R package phytools^69^. For birds and mammals, 1000 phylogenetic trees were available representing the phylogenetic uncertainties^64,65^. We considered the phylogenetic uncertainties by calculating the eigenvectors as average the eigenvector obtained for each phylogeny.

It is important to note that, compared to traditional imputation procedures, we aimed to characterize the position of species in the corresponding trait space rather than estimating the values of the original traits (see ‘World and realm functional spectra’ below). Thus, we tested the accuracy of our trait imputation procedure by comparison of the position of the species with complete trait values in the functional space (real position of the species) with the position of the same species for which we had artificially removed traits in the different dimensions of the functional space. We estimated the performance of the imputation using the normalized root mean square error (NRMSE), which expresses the average distance between real and imputed positions of species as a proportion of the range of values of species in the corresponding dimension. To do it, we artificially removed 10% of trait values from a subset of species with complete information and selected one random species with incomplete information and superimposed its pattern of missing values. Thus, we kept constant the pattern of missing values as the one in the original dataset. We performed the phylogenetically-informed imputation procedure as described above using the entire dataset consisting of all the species with non-complete trait information and the species with complete trait information (i.e. including 90% of species with complete trait information and 10% of species with complete trait information plus artificial missing values). This way, the ratio between missing and complete data was higher in the simulations than in the original dataset, therefore ensuring a conservative test of the quality of our imputation procedure. In addition, we repeated this examination 100 times for each taxonomic group and measured the standard error of the NRMSE, which was always <0.5% demonstrating that these repetitions gave similar results (Supplementary Table 2). Finally, we imputed traits to project species onto the functional space based on the full dataset.

#### Conservation status of the species

We collected the conservation status of species from the IUCN Red List (version 2020-3^15^) using the R package ‘rredlist’^70^. For each taxonomic group, we used the IUCN classes: CR: critically endangered; EN: Endangered; VU: Vulnerable; NT: Near Threatened; LC: Least Concern and DD: Data Deficient. In total, we retrieved the status for 6388 mammals, 11,158 birds, 8295 reptiles, 7167 amphibians and 20,295 fish species (including marine species).

Matching occurrences, functional traits and IUCN databases. Taxonomies from all the used sources (trait databases, spatial occurrences, phylogenies and IUCN Red List^15^), were standardized using the R packages ‘taxize’^71^. All names were resolved against the GBIF Backbone Taxonomy. Among taxonomic groups, the proportion of species described by spatial and functional trait databases varied between 53% for reptiles (5689 functionally described species out of 10,783 species with geographic distributions) and 74% for mammals (4408 species with trait information out of 5926 species). At the realm scale, the proportion of species with trait information within each taxonomic group was congruent with the proportion of species observed using only spatial data (Supplementary Table 1, Supplementary Data 1, Supplementary Table 3, and Supplementary Fig. 1). Matching species occurrences, functional traits and IUCN status revealed that vertebrates had relatively high information coverage, ranging from 44% for reptiles and freshwater fishes to 74% for mammals (Supplementary Table 1).

To assess the relevance of the subset of species used in the analyses compared to all species, we quantified how much the functional traits of threatened species differ from the functional traits of non-threatened species, both in terms of (1) realm coverage and (2) trait distortion. (1) We used chi-squared tests between the occurrence dataset (considered as the most complete database), the functional dataset and the IUCN Red List dataset (Supplementary Table 3). We compared the proportion of species informed by IUCN in each realm to the expected proportion of species based on the species spatially informed (chi-squared test, see Supplementary Table 3). For each realm, we quantified the standard deviation from the expected number of species with spatial occurrences. (2) We measured to what extent these differences might affect the shape of the functional space for each realm. For that, we calculated the overlap between the functional space built with all species spatially informed in each realm and the functional space built with the subset of species evaluated by IUCN Red List (Supplementary Table 5).

We used the IUCN Red List database only to assess the loss of functional diversity. Otherwise, to describe the taxonomic and functional diversity patterns, we used species that are both spatially and functionally informed, regardless of the information about their conservation status.

### World and realm functional spectra

The construction of the spectra of each taxonomic group was similar to the procedure followed in ref. ^11^. Briefly, we identified the main axes of functional trait variation by performing principal component analyses (PCA) on the log-transformed and scaled functional traits of each taxonomic group. Spectra were built using all species for which we had trait information. Two dimensions were needed for all taxonomic groups but freshwater fish, for which four dimensions were needed (Supplementary Fig. 3 and see ref. ^11^ for details). We estimated the probabilistic distribution of the species within the functional spaces using all species with spatial and functional information by performing multivariate kernel density estimations with the ‘TPD’^26^ R package. Although TPD functions are continuous, in order to perform operations with them it is more practical to divide the functional space into a D-dimensional grid composed of many equal-sized cells (we divided the 2-dimensional spaces into 40,000 cells, 200 per dimension, and the 4-dimensional space in 810,000 cells, 30 per dimension). Then, the value of the TPD function is estimated for each cell. The value of the TPD function in a given point of the space reflects the density of species in that particular area of the space (i.e. species with similar functional traits). The kernel for each species was a multivariate normal distribution centred in the coordinates of the species in the functional space and bandwidth chosen using unconstrained bandwidth selectors from the ‘Hpi’ function in the ‘ks’^72^ package (see details in ref. ^11^). To facilitate comparisons, the kernel for each species was kept constant for generating the functional space of each biogeographic realm.

### Extinctions of threatened species

To test how functional diversity can be affected by species loss at different biogeographic realms, we simulated the loss of threatened species in a progressive framework according to the IUCN status of the species^15^. The species classified among the most threatened species category (i.e. critically endangered (CR)) have a higher risk of extinction than the species with a lower threat (vulnerable (VU), endangered (EN), near threatened (NT)). We started removing the species with a higher risk of extinction (-CR), then we removed successively the species with lower threatened risks (-EN: CR and EN removed, -VU: CR, EN and VU removed, -NT: CR, EN, VU and NT removed). These simulations mimicked a gradient of extinction risk from a scenario where only the most endangered species went extinct to a more dramatic scenario where all threatened species (including the NT species) went extinct. For convenience, we named the scenarios according to the least threatened category considered. We also ran the last scenario considering the extinction of all threatened and near-threatened species plus all the data deficient species (i.e. -DD: CR, EN, VU, NT and DD). This scenario considers the eventuality that all data deficient species are threatened and therefore represents an extreme scenario compensating for potential incompleteness in species threat evaluation by the IUCN.

We estimated in each biogeographic realm how functional diversity will change in case of extinction of threatened species. For that, in each functional space, we estimated a TPD function considering all the species assessed by IUCN^15^, and another TPD function after removing the species classified as threatened. Whereas the TPD functions of all species assessed by IUCN reflect the current spectra, the TPD functions after removing threatened species reflect the potential spectra if threatened species go extinct. TPD functions are probability density functions so that they integrate to 1 across the whole functional space, a property that permits the comparison of different TPD functions^18^. We applied a quantile threshold of 99% to reduce the potential effect of outliers on the estimation of the amount of functional space occupied by the different spectra. After thresholding, the TPD functions were rescaled, and the probabilities were expressed in terms of quantiles to ease the interpretability of the results^11,18^.

We represented the impact of simulated extinctions by subtracting in each point of the functional space, the quantile value of the TPD function after removing threatened species from the quantile value of the TPD function of IUCN-assessed species. Negative values in this index indicate a decrease in the relative abundance of the trait values corresponding to a functional space cell and vice versa. To quantify how much the functional spectra of each group will change after extinctions, we estimated for each cell the absolute value of this quantile difference and averaged these values across cells. With this approach, we could also characterize which functional space cells become empty after extinctions (lost space; expressed as a proportion of the total space occupied by the IUCN-assessed species spectra).

### Biodiversity indices

Taxonomic diversity was calculated as the number of species in each biogeographic realm (i.e. taxonomic richness, TRic) and endemicity was calculated for each realm as the proportion of the species occurring only in that realm. Functional diversity was measured as the amount of functional space occupied by the spectra (i.e. functional richness, FRic). We also calculated taxonomic (TDiss) and functional dissimilarity (FDiss) between biogeographic realms using the Jaccard dissimilarity index (for taxonomic diversity) and overlap-based dissimilarity (for functional diversity) as implemented in the ‘betapart’ and ‘TPD’ R packages, respectively^26,73^. The Jaccard dissimilarity index measures, for two assemblages, the proportion of unique species and ranges from 0 (identical species) to 1 (completely different species)^74^. The overlap-based dissimilarity between two assemblages reflects the degree of overlap between the probabilistic distributions of species in the functional space between the two assemblages and ranges from 0 (complete overlap) to 1 (no overlap)^26^.

### Null models

For each biogeographic realm, we compared the distribution of species within the functional space with a null model where the same number of species were randomly selected from the world’s pool of species. For each taxonomic group and realm, we drew 999 simulated assemblages and compared the functional richness of those 999 assemblages to the observed FRic. We then calculated standardized effect sizes (SES) as the difference between the observed value and mean of the simulated ones standardized by the standard deviation of the simulated values. We ranked the observed FRic against the simulated FRic and calculated the *P*-values to indicate the statistical significance of the rank. *P*-values higher than 0.975 indicate that the observed FRic is significantly higher than expected, *P*-values lower than 0.025 indicate that the observed FRic is significantly lower than expected given the number of species.

To test whether the SES values were correlated to the species richness in each realm, we performed linear regression models between the SES and the number of species in each realm for each taxonomic group. A negative relationship shows that species-rich assemblages tend to be functionally clustered while species-poor assemblages tend to be overdispersed. A positive relationship shows the opposite pattern, species-rich assemblages will host even more functionally diverse species while species-poor assemblages are functionally clustered.

To assess if the impacts of potential extinctions in each realm are different from what would be expected if extinction risk is not related to species’ traits, we also compared the observed changes in functional diversity to a null model where the extinct species are randomly selected within the realm’s pool of species. For each scenario of extinction risk, we compared the loss of functional diversity to 999 losses of functional diversity where the species traits of threatened species were randomly selected among the realm pool of species. This strategy allowed us to ascertain whether losing threatened species reduces more or less than expected the functional spectra of the different taxonomic groups. For each scenario, we created 999 TPD functions simulating cases in which the same number of species were lost at random from the total set of IUCN-assessed species from the corresponding realm (i.e. including both non-threatened and threatened species rather than only threatened ones). We performed similar comparisons between each of these simulated spectra and the spectra of the IUCN-assessed species and calculated the *P*-values to indicate the statistical significance of the rank.

For each taxonomic group, we tested whether the SES values of each biogeographic realm were significantly different from each other using multiple pairwise comparison tests. This allowed us to compare the differences between multiple normal distributions. To obtain distributions from SES values, we used a bootstrapping procedure where we calculated 99 SES values from a sample of 99 FRic values among the 999 simulated assemblages. We used this procedure for both the SES of the current FRic patterns and the changes in FRic under the loss of threatened species for each scenario. Pairwise comparisons results are shown by a compact letter display of all pairwise comparisons with a significance level at 5%.

### Reporting summary

Further information on research design is available in the Nature Research Reporting Summary linked to this article.

## Supplementary information

Supplementary Information

Description of Additional Supplementary Files

Supplementary data 1

Supplementary data 2

Reporting Summary

## Data Availability

The data used in this study are available in the database under accession code in Figshare (https://figshare.com/s/f076a046963c6f782f8d). AmphiBIO are available at 10.1038/sdata.2017.123. IUCN Red List data are available at http://www.iucnredlist.org. IUCN spatial database available at https://www.iucnredlist.org/resources/spatial-data-download. Mammal data are available at https://www.mammaldiversity.org/. AMIOTE data are available at 10.1890/15-0846R.1. Fishbase is available at http://www.fishbase.org. WWF data used are available at www.worldwildlife.org/WildFinder. GBIF data are available at 10.15468/dl.xabdgp; 10.15468/dl.ixznsa; 10.15468/dl.pdvdl4; 10.15468/dl.j7zy2r. Source data are provided with this paper.

## Source data

SourceData

## Code availability

All data sets, as well as R codes involved in data processing, statistical analysis, and plotting of the results (including a Source data file), are available in Figshare (https://figshare.com/s/f076a046963c6f782f8d).
