# Supplementary material for: Extinction of threatened vertebrates will lead to idiosyncratic changes in functional diversity across the world
Source: Nat Commun. 2021 Aug 27;12:5162. doi: 10.1038/s41467-021-25293-0 (PMC8397725; doi:10.1038/s41467-021-25293-0)
Supplement: Supplementary file 1 — Supplementary Information [file 41467_2021_25293_MOESM1_ESM.pdf]

Supplementary Table 1. The number of species informed for spatial, traits and the IUCN Red List databases.

Database	Taxonomic group	Afrotropical	Australian	Nearctic	Neotropical	Indo-Malay	Palearctic	World
Spatial	Mammals	1508	880	583	1721	1222	1023	5926
	Birds	3166	2932	1224	4750	3176	2124	12863
	Reptiles	2288	1932	708	3659	2121	1122	10783
	Amphibians	1235	744	419	4103	1269	500	7864
	Freshw. fish	2919	627	936	5248	2629	1396	13008
	All groups	11116	7115	3870	19481	10417	6165	50444
Traits	Mammals	1101 (73%)	747 (85%)	470 (81%)	1221 (71%)	951 (78%)	821 (80%)	4408 (74%)
	Birds	2083 (66%)	1842 (63%)	916 (75%)	3531 (74%)	2005 (63%)	1456 (69%)	8564 (67%)
	Reptiles	1243 (54%)	1271 (66%)	430 (61%)	1813 (50%)	889 (42%)	646 (58%)	5689 (53%)
	Amphibians	980 (79%)	597 (80%)	303 (72%)	2753 (67%)	841 (66%)	323 (65%)	5548 (71%)
	Freshw. fish	2340 (80%)	441 (70%)	796 (85%)	3844 (73%)	1915 (73%)	1104 (79%)	9771 (75%)
	All groups	7747 (70%)	4898 (69%)	2915 (75%)	13162 (68%)	6601 (63%)	4350 (71%)	33980 (67%)
IUCN Red List	Mammals	1084 (72%)	739 (84%)	467 (80%)	1215 (71%)	946 (77%)	814 (80%)	4364 (74%)
	Birds	1927 (61%)	1696 (58%)	840 (69%)	3406 (72%)	1808 (57%)	1336 (63%)	7986 (62%)
	Reptiles	905 (40%)	1121 (58%)	397 (56%)	1652 (45%)	660 (31%)	486 (43%)	4769 (44%)
	Amphibians	958 (78%)	532 (72%)	284 (68%)	2670 (65%)	765 (60%)	285 (57%)	5272 (67%)
	Freshw. fish	1920 (66%)	367 (59%)	759 (81%)	1104 (21%)	1468 (56%)	627 (45%)	5785 (44%)
	All groups	6794 (61%)	4455 (63%)	2747 (71%)	10047 (52%)	5647 (54%)	3548 (58%)	28176 (56%)

For traits and the IUCN Red List, the values in parentheses represent the percentage of species with spatial occurrences. The number of spatial species informed is considered as the most accurate number of species available.

Supplementary Table 2. Performance of the trait imputation procedure.

	PC1	PC2	PC3	PC4
Mammals	2.24 ± 0.06%	0.95 ± 0.02%	—	—
Birds	2.90 ± 0.20%	3.17 ± 0.19%	—	—
Reptiles	3.19 ± 0.15%	2.39 ± 0.14%	—	—
Amphibians	8.99 ± 0.38%	8.78 ± 0.33%	—	—
Freshwater fishes	5.86 ± 0.17%	3.31 ± 0.11%	15.75 ± 0.40%	11.87 ± 0.32%

For each taxonomic group, we simulated missing traits among the subset of species with complete traits. For this, we simulated missing traits for 10% of the species with complete traits in each taxonomic group. To each of these randomly selected species we assigned the structure of missing values of a random species from the subset of species with missing trait values. We then combined the three subsets of species (90% of species with complete trait information, 10% of species with complete trait information plus artificial missing values and all species with non-complete trait information) into a single dataset in which we performed the phylogenetically-informed imputation procedure (see details in Method). We then used the imputed dataset to predict the position of all species in the functional space using the PCA performed on the whole dataset for each taxonomic group. Finally, only for the species with artificially added missing values, we estimated, for each dimension of the functional space, the difference between the observed position of the species in the functional space (i.e. using observed traits) and the position calculated after artificial removal of traits and trait imputation. We expressed these differences in terms of normalised root mean square error (NRMSE), measured as percentage of the range of trait values in the corresponding PC axis. We repeated this procedure 100 times for each taxonomic group. The table contains, for each taxonomic group and dimension of its functional space, the mean ± standard error of the NRMSE values across the 100 repetitions.

Supplementary Table 3. Comparison between the proportion of functionally-informed species or the IUCN Red List and species with spatial occurrences in each biogeographic realm.

Database	Taxonomic group	χ^2	df	P value	Afrotropical	Australian	Nearctic	Neotropical	Indo-Malay	Palearctic
Traits	Mammals	20.9	5	<0.001	-1.8	3.0	1.2	-3.1	0.6	1.5
	Birds	61.3	5	<0.001	-1.8	-3.8	3.0	6.1	-3.8	0.3
	Reptiles	129.7	5	<0.001	0.8	8.3	2.8	-3.6	-7.9	2.1
	Amphibians	36.4	5	<0.001	4.2	3.5	0.6	-3.2	-1.8	-1.5
	Freshw. fish	29.8	5	<0.001	3.0	-1.6	3.3	-2.8	-2.0	1.4
IUCN RED LIST	Mammals	20.7	5	<0.001	-2.0	2.9	1.2	-2.9	0.7	1.5
	Birds	95.5	5	<0.001	-2.0	-4.1	2.4	8.4	-5.1	-0.3
	Reptiles	200.6	5	<0.001	-3.7	10.0	4.9	1.1	-10.0	-0.4
	Amphibians	41.1	5	<0.001	5.2	1.8	0.3	-1.5	-2.9	-2.7
	Freshw. fish	1302.6	5	<0.001	18.4	5.0	16.8	-33.3	8.8	-0.3

We used a chi-squared (χ^2) test in order to compare proportions. For each realm, we reported the standard deviation from the expected number of species with spatial occurrences**.** Negative values mean that the number of functionally-informed or IUCN Red List species is lower than expected if the spatial database were equally informed between realms, positive values mean that the number of functionally-informed or IUCN Red List species is higher than expected.

Supplementary Table 4. Number of species with known status according to IUCN Red List.

	CR	EN	VU	NT	DD	LC	NE
Mammals	155	383	409	289	439	2759	241
Birds	131	290	531	659	23	6387	888
Reptiles	125	339	278	262	434	3424	1345
Amphibians	488	763	524	317	867	2419	870
Freshw. fish	217	351	413	246	923	4234	4199

For each taxonomic group, the values indicate the number of species informed in each IUCN class and the number of species that have been not evaluated (NE). CR: critically endangered; EN: Endangered; VU: Vulnerable; NT: Near Threatened; LC: Least Concern; NE: Not Evaluated by IUCN.

Supplementary Table 5. Similarity between functional spectra considering all species and functional spectra considering only species with known status according to IUCN Red List.

	World	Afrotropical	Australian	Nearctic	Neotropical	Indo-Malay	Palearctic
Mammals	98.17	98.75	99.19	99.41	99.59	99.56	99.28
Birds	98.34	97.32	96.56	95.55	98.36	96.13	96.46
Reptiles	96.46	90.62	96.16	95.85	96.35	91.57	91.40
Amphibians	98.10	98.50	93.56	95.30	98.40	95.70	93.15
Freshw. fish	73.99	86.24	84.18	95.59	50.50	82.10	65.10

Species with known status according to IUCN Red List encompass CR, EN, VU, NT, DD and LC. The values indicate the probabilistic percentage of overlap between the respective TPD function of all species and the subset of species with known IUCN status.

Supplementary Table 6. Current taxonomic and functional diversity of the vertebrates across the six biogeographic realms.

A) Observed

Taxonomic group	Index	Afrotropical	Australian	Nearctic	Neotropical	Indo-Malay	Palearctic	Rho
Mammals	TRic	24.97	16.94	**10.66**	**27.72**	21.57	18.62	
	FRic	**85.96**	71.40	**62.36**	75.25	80.35	76.50	$\mathbf{0.79}^{***}$
Birds	TRic	24.32	21.51	**10.70**	**41.23**	23.41	17.00	
	FRic	77.44	79.58	**72.19**	**81.48**	79.03	74.00	$\mathbf{0.86}^{***}$
Reptiles	TRic	21.85	22.34	**7.56**	**31.87**	15.63	11.36	
	FRic	**72.89**	66.90	**47.65**	63.79	63.18	56.25	$\mathbf{0.86}^{***}$
Amphibians	TRic	17.66	10.76	**5.46**	**49.62**	15.16	5.82	
	FRic	63.95	**45.59**	58.64	**83.88**	50.16	45.88	0.75^{ns}
Freshw. fish	TRic	23.95	**4.51**	8.15	**39.34**	19.60	11.30	
	FRic	35.43	**12.04**	15.28	**55.66**	36.84	23.23	$\mathbf{0.96}^{***}$

B) Standardized effect size

Taxonomic group	Realm	SES	MeanRd	CI025Rd	CI975Rd	pvalues	Nreps
Mammals	Afrotropical	1.14	83.31	78.61	88.00	0.88	999
	**Australian**	**-3.02**	**78.55**	**74.22**	**83.24**	**<0.001**	**999**
	**Nearctic**	**-4.22**	**72.67**	**67.60**	**77.18**	**<0.001**	**999**
	**Neotropical**	**-4.48**	**84.71**	**80.63**	**88.73**	**<0.001**	**999**
	Indo-Malay	-0.53	81.63	76.71	86.38	0.29	999
	Palearctic	-1.43	79.87	75.46	84.83	0.07	999
Birds	Afrotropical	-1.10	79.47	76.05	83.00	0.14	999
	Australian	0.96	77.77	74.09	81.48	0.82	999
	**Nearctic**	**2.81**	**66.55**	**62.55**	**70.37**	**>0.999**	**999**
	**Neotropical**	**-3.53**	**87.28**	**84.03**	**90.45**	**<0.001**	**999**
	Indo-Malay	0.05	78.94	75.50	82.45	0.51	999
	Palearctic	-0.10	74.21	70.39	78.21	0.45	999
Reptiles	Afrotropical	-0.44	74.04	69.02	79.17	0.34	999
	**Australian**	**-3.15**	**74.46**	**69.75**	**78.95**	**<0.001**	**999**
	**Nearctic**	**-3.10**	**56.02**	**50.94**	**61.52**	**<0.001**	**999**
	**Neotropical**	**-7.08**	**80.69**	**76.06**	**85.53**	**<0.001**	**999**
	**Indo-Malay**	**-1.87**	**68.21**	**63.32**	**73.54**	**0.02**	**999**
	**Palearctic**	**-2.33**	**62.75**	**57.58**	**68.05**	**<0.001**	**999**
Amphibians	**Afrotropical**	**-4.60**	**74.94**	**70.44**	**79.90**	**<0.001**	**999**
	**Australian**	**-9.43**	**68.23**	**63.73**	**73.02**	**<0.001**	**999**
	Nearctic	-0.09	58.84	54.59	63.48	0.50	999
	**Neotropical**	**-2.71**	**89.37**	**85.30**	**93.24**	**<0.001**	**999**
	**Indo-Malay**	**-9.37**	**72.88**	**68.29**	**77.59**	**<0.001**	**999**
	**Palearctic**	**-5.81**	**59.96**	**55.11**	**64.70**	**<0.001**	**999**
Freshw. fish	**Afrotropical**	**-14.51**	**43.32**	**42.29**	**44.36**	**<0.001**	**999**
	Australian	-0.89	12.27	11.77	12.77	0.19	999
	**Nearctic**	**-13.21**	**19.87**	**19.20**	**20.58**	**<0.001**	**999**
	**Neotropical**	**-5.99**	**59.30**	**58.05**	**60.51**	**<0.001**	**999**
	**Indo-Malay**	**-1.95**	**37.85**	**36.84**	**38.87**	**0.03**	**999**
	**Palearctic**	**-5.67**	**25.51**	**24.72**	**26.26**	**<0.001**	**999**

Taxonomic diversity was calculated as the number of species in each biogeographic realm (i.e. taxonomic richness, TRic) and functional diversity was measured as the functional richness (FRic, amount of functional space occupied by the spectra). A) corresponds to the current composition of species. Values are given in percentage of the world TRic and FRic for each taxonomic group. The least and the most diverse realms are indicated in bold. The correlation between TRic and FRic has been tested using a two-sided Spearman's rank correlation test for each taxonomic group. We reported the coefficient of correlation (Rho) and its associate P-value (***: $P < 0.001$; ns: $P > 0.05$). B) For each biogeographic realm, we compared the distribution of species within the functional space with a null model where the same number of species were randomly selected from the world's pool of species. For each taxonomic group and realm, we drew 999 simulated assemblages and compared the functional richness of those 999 assemblages (Nreps) to the observed functional richness using two-tailed t-tests. We then calculated standardized effect sizes (SES) as the difference between the observed value and mean of the simulated ones standardized by the standard deviation of the simulated values. P values were calculated by ranking the observed changes among the simulated ones. Statistical parameters of the 999 simulated FRic is given: mean (MeanRD) and confidence interval at 95% (CI025Rd, CI975Rd). Values are given in percentage of the world FRic for each taxonomic group. Significant results (p-values < 0.025 and > 0.975) are indicated in bold.

Supplementary Table 7. The number of species occurring in only one or more realms.

A) All species with spatial occurrences

Taxonomic group	1 Realm	2 Realms	3 Realms	4 Realms	5 Realms	6 Realms
Mammals	5039	776	92	11	2	2
Birds	9496	2531	530	157	52	56
Reptiles	9727	921	49	5	0	5
Amphibians	7450	373	12	2	1	0
Freshw. fish	12344	595	54	8	3	2

B) The subset of species with spatial occurrences and functionally informed

Taxonomic group	1 Realm	2 Realms	3 Realms	4 Realms	5 Realms	6 Realms
Mammals	3631	672	90	11	2	2
Birds	6254	1644	430	131	39	47
Reptiles	5124	524	36	3	0	0
Amphibians	5313	223	10	2	0	0
Freshw. fish	9178	533	49	7	3	1

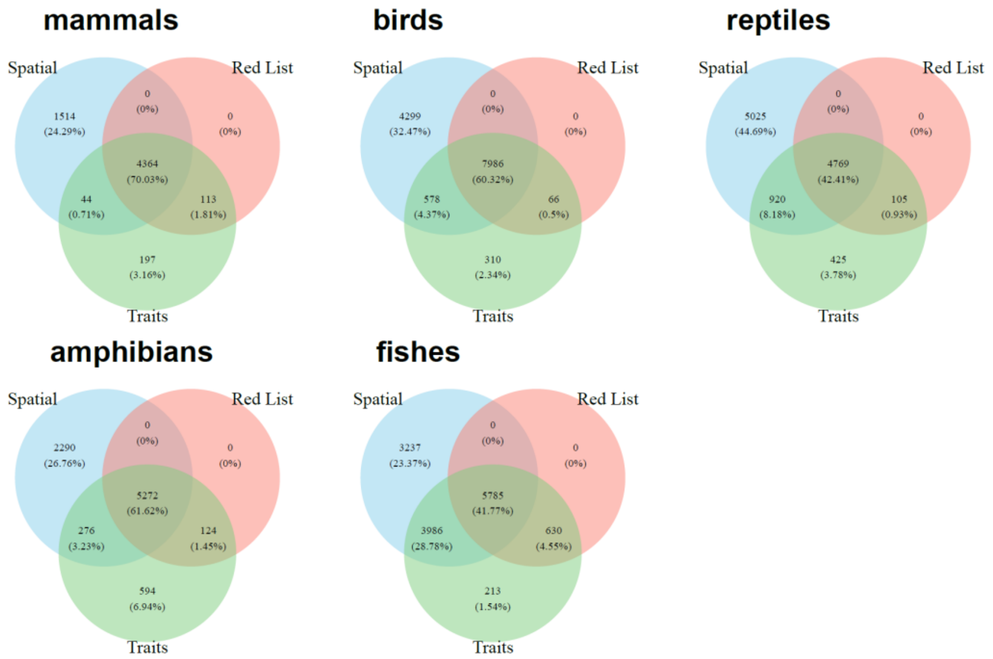

Supplementary Fig 1. Proportion of species shared by each database for the world. For each taxonomic group is indicated the number and the proportion of species informed in each database and in combination (see also Supplementary Table 1, Supplementary Table 2). Note that the subset of species informed for Spatial and Traits were used to analyse the taxonomic diversity and functional diversity patterns, and the subset of species informed for all three databases was used to analyse the changes in taxonomic diversity and functional diversity due to the simulated removal of threatened species.

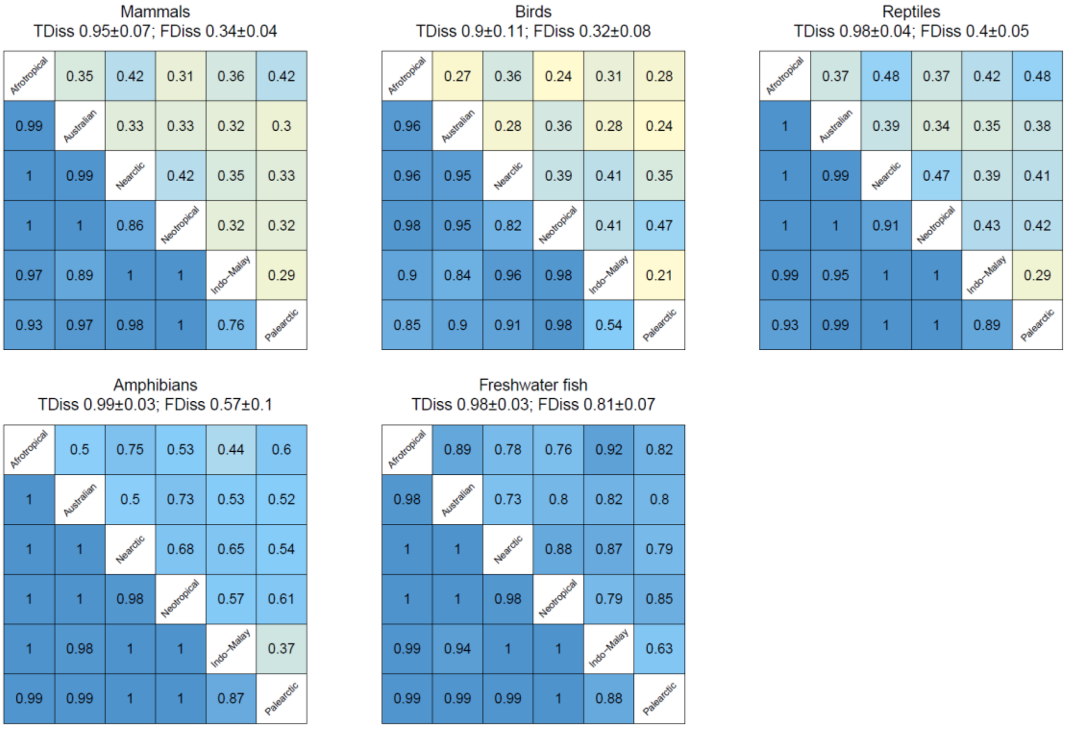

Supplementary Fig 2. Taxonomic and functional dissimilarity between the six biogeographic realms. The matrix for each group shows the distance (Jaccard dissimilarity index for taxonomic and overlap for functional, see details in the Methods) between the six biogeographic realms considering the taxonomic (lower triangle of the matrix) and functional (upper triangle of the matrix) diversity. One panel for each taxonomic group. The colour gradient (yellow – blue) depicts the value of the dissimilarity from 0 (i.e. assemblages completely different, yellow) to 1 (i.e. assemblages completely similar, blue). Each taxonomic group is indicated with the mean and SD of the taxonomic (TDiss) and functional dissimilarity (FDiss) among the biogeographic realms.

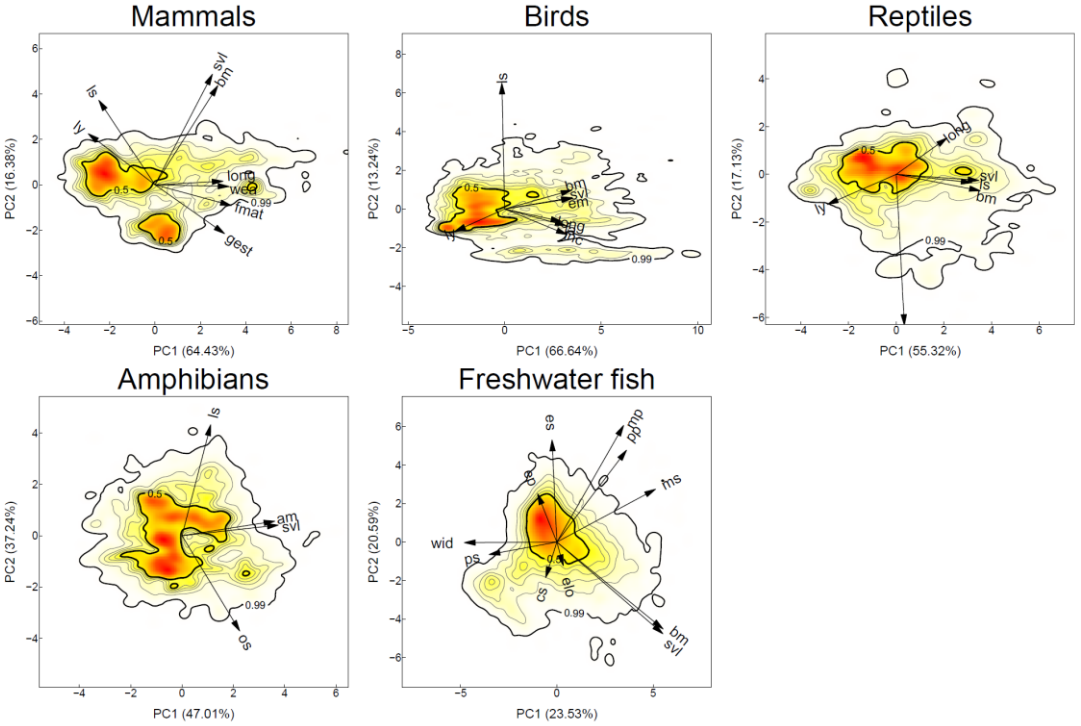

Supplementary Fig 3. Global functional spectra for the six taxonomic groups. Probabilistic species distributions in the spaces are defined by the two first principal components of PCA considering different functional traits for each group (see Table 1 for definitions of each functional trait). Arrows indicate the direction and weighting of each trait in the PCA (see Methods for description of each trait, and Table 1 for the meaning of the traits). The colour gradient (red-yellow-white) depicts different density of species in the defined space (red areas are more densely populated). Thick contour lines indicate the 0.5 (hotspots, see main text) and 0.99 quantiles, and thinner ones indicate quantiles 0.6, 0.7, 0.8 and 0.9. Adapted from ref.[1].

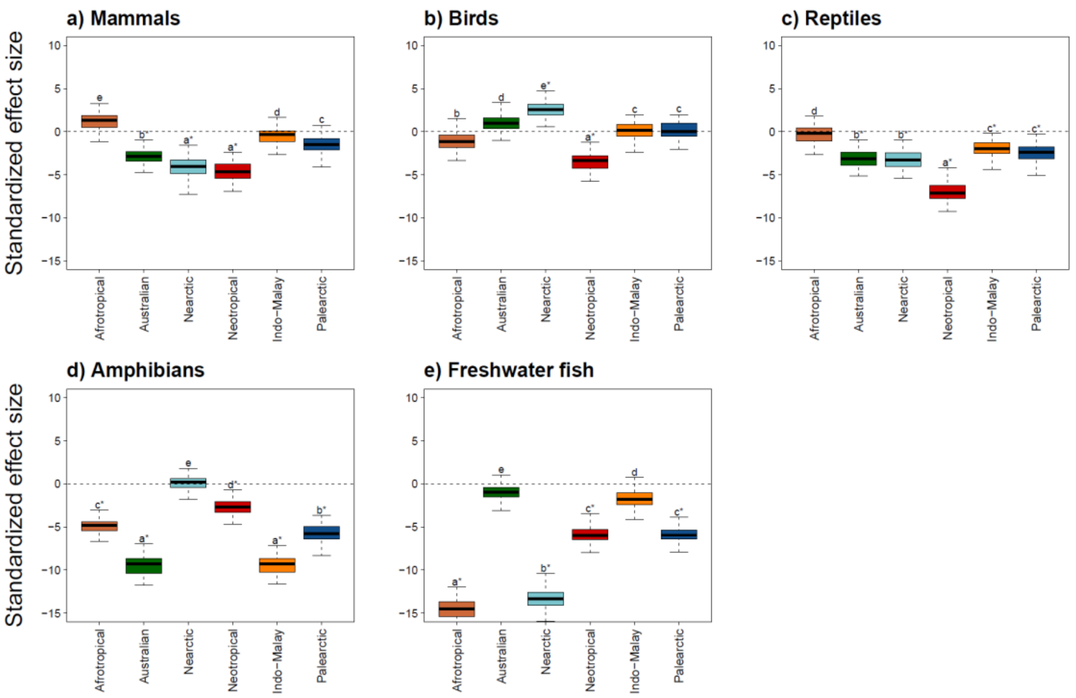

Supplementary Fig 4. Standardized effect size (SES) distribution for the six taxonomic groups in the six biogeographic realms. For each taxonomic group (panels a-e), we tested whether the SES values of the functional richness of each biogeographic realm were significantly different from each other using multiple pairwise comparison tests. SES distributions were obtained using a bootstrapping procedure (n=1000 repetitions, see the Methods for details). We used this procedure for the SES of the current FRic patterns. We show for each group a compact letter display of all pairwise comparisons with a significance-level at 5%. The SES significantly different than expected under null models is identified by an asterisk.

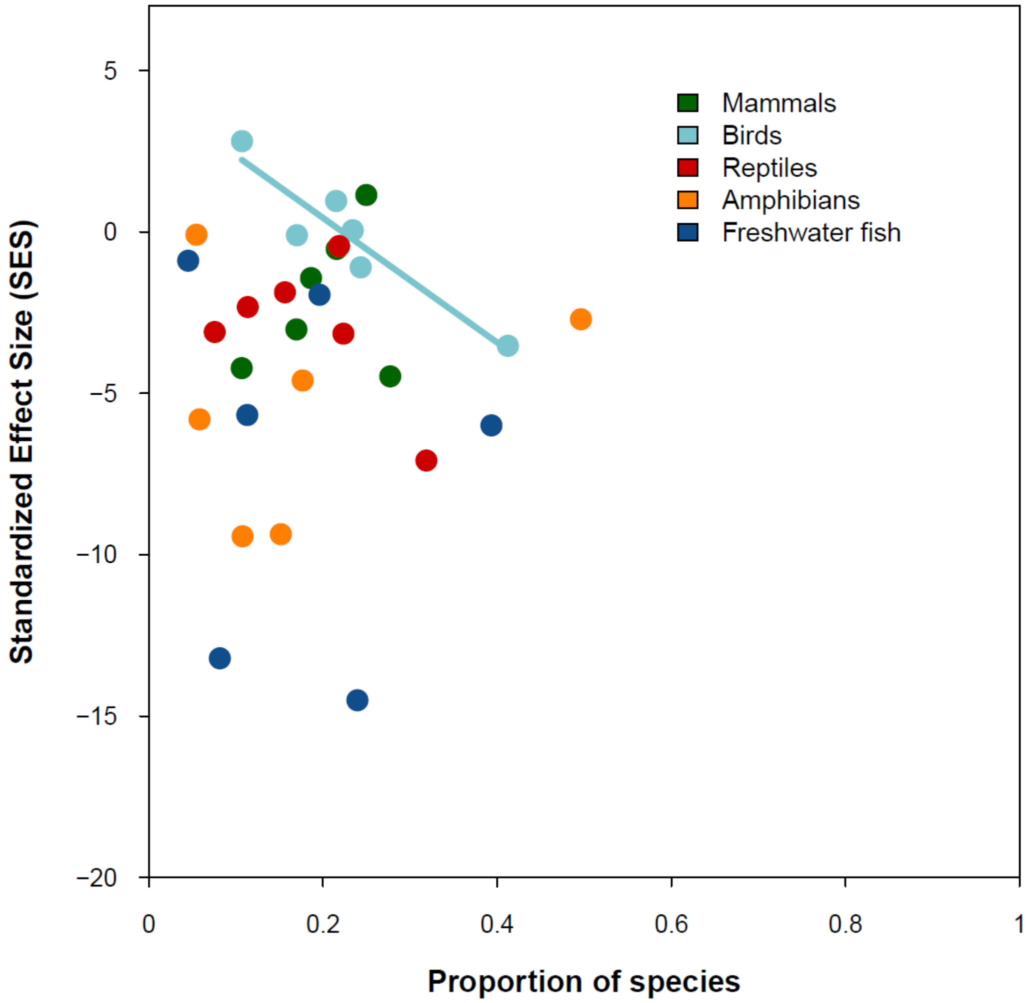

Supplementary Fig 5. Relationship between the standardized effect size (SES) and the proportion of species in each realm for each taxon. We performed linear regression models between the SES of functional richness and the proportion of species in each realm for each taxon. Each point represents a realm. A linear regression line represents a significant regression. For each taxonomic group and realm, we drew 999 simulated assemblages. We then calculated SES as the difference between the observed value and mean of the simulated ones standardized by the standard deviation of the simulated values.

A) Mammals

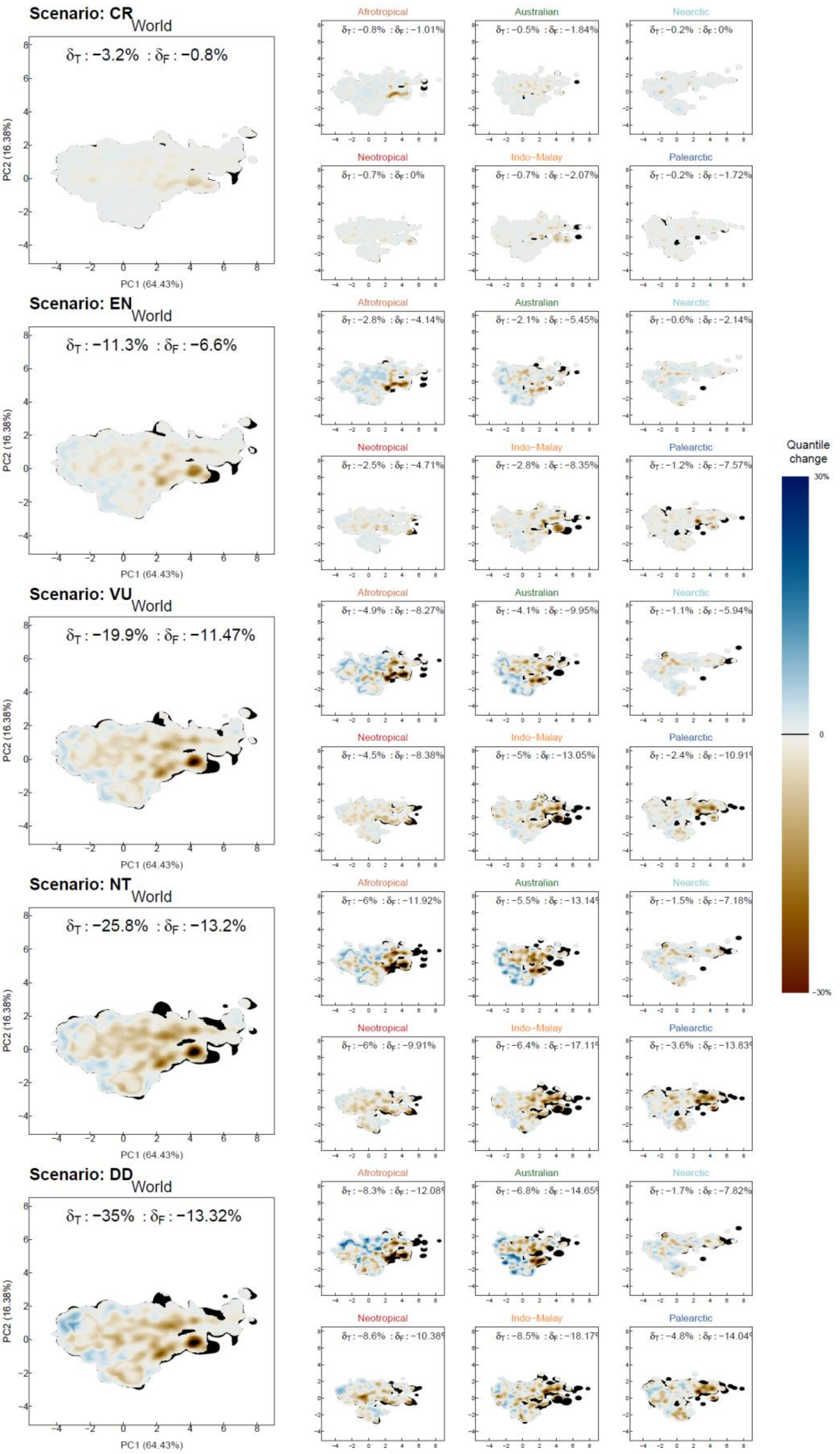

Scenario: CR
World
δT : −3.2% : δF : −0.8%
Afrotropical
δT : −0.8% : δF : −1.01%
Australian
δT : −0.5% : δF : −1.84%
Nearctic
δT : −0.2% : δF : 0%
Neotropical
δT : −0.7% : δF : 0%
Indo−Malay
δT : −0.7% : δF : −2.07%
Palearctic
δT : −0.2% : δF : −1.72%
Scenario: EN
World
δT : −11.3% : δF : −6.6%
Afrotropical
δT : −2.8% : δF : −4.14%
Australian
δT : −2.1% : δF : −5.45%
Nearctic
δT : −0.6% : δF : −2.14%
Neotropical
δT : −2.5% : δF : −4.71%
Indo−Malay
δT : −2.8% : δF : −8.35%
Palearctic
δT : −1.2% : δF : −7.57%
Scenario: VU
World
δT : −19.9% : δF : −11.47%
Afrotropical
δT : −4.9% : δF : −8.27%
Australian
δT : −4.1% : δF : −9.95%
Nearctic
δT : −1.1% : δF : −5.94%
Neotropical
δT : −4.5% : δF : −8.38%
Indo−Malay
δT : −5% : δF : −13.05%
Palearctic
δT : −2.4% : δF : −10.91
Scenario: NT
World
δT : −25.8% : δF : −13.2%
Afrotropical
δT : −6% : δF : −11.92%
Australian
δT : −5.5% : δF : −13.14
Nearctic
δT : −1.5% : δF : −7.18%
Neotropical
δT : −6% : δF : −9.91%
Indo−Malay
δT : −6.4% : δF : −17.11
Palearctic
δT : −3.6% : δF : −13.83
Scenario: DD
World
δT : −35% : δF : −13.32%
Afrotropical
δT : −8.3% : δF : −12.08
Australian
δT : −6.8% : δF : −14.65
Nearctic
δT : −1.7% : δF : −7.82%
Neotropical
δT : −8.6% : δF : −10.38
Indo−Malay
δT : −8.5% : δF : −18.17
Palearctic
δT : −4.8% : δF : −14.04
PC1 (64.43%)
PC2 (16.38%)
Quantile change
30%
0
−30%

B) Birds

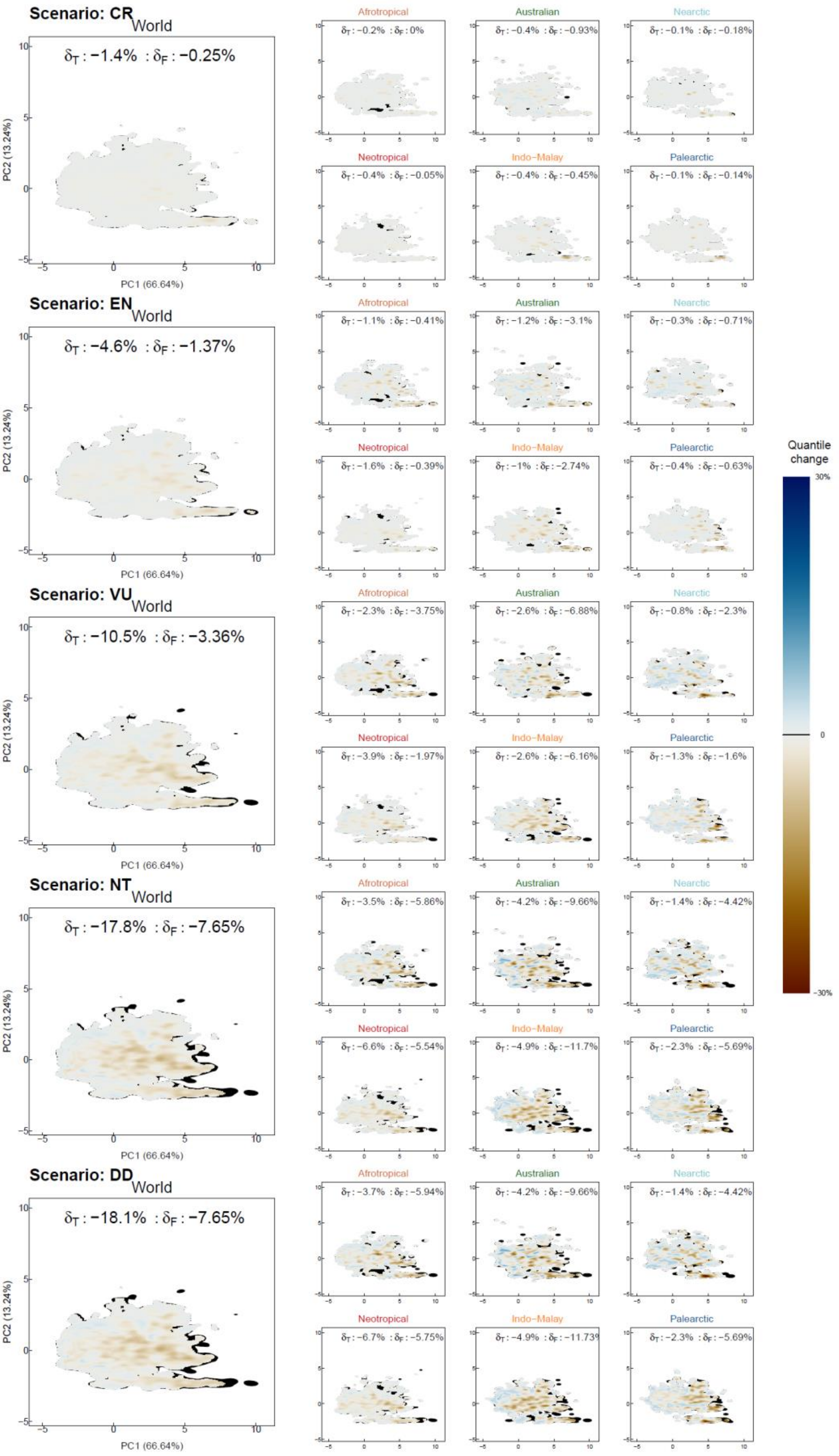

Scenario: CR
World
δT : −1.4% : δF : −0.25%
Afrotropical
δT : −0.2% : δF : 0%
Australian
δT : −0.4% : δF : −0.93%
Nearctic
δT : −0.1% : δF : −0.18%
Neotropical
δT : −0.4% : δF : −0.05%
Indo−Malay
δT : −0.4% : δF : −0.45%
Palearctic
δT : −0.1% : δF : −0.14%
Scenario: EN
World
δT : −4.6% : δF : −1.37%
Afrotropical
δT : −1.1% : δF : −0.41%
Australian
δT : −1.2% : δF : −3.1%
Nearctic
δT : −0.3% : δF : −0.71%
Neotropical
δT : −1.6% : δF : −0.39%
Indo−Malay
δT : −1% : δF : −2.74%
Palearctic
δT : −0.4% : δF : −0.63%
Scenario: VU
World
δT : −10.5% : δF : −3.36%
Afrotropical
δT : −2.3% : δF : −3.75%
Australian
δT : −2.6% : δF : −6.88%
Nearctic
δT : −0.8% : δF : −2.3%
Neotropical
δT : −3.9% : δF : −1.97%
Indo−Malay
δT : −2.6% : δF : −6.16%
Palearctic
δT : −1.3% : δF : −1.6%
Scenario: NT
World
δT : −17.8% : δF : −7.65%
Afrotropical
δT : −3.5% : δF : −5.86%
Australian
δT : −4.2% : δF : −9.66%
Nearctic
δT : −1.4% : δF : −4.42%
Neotropical
δT : −6.6% : δF : −5.54%
Indo−Malay
δT : −4.9% : δF : −11.7%
Palearctic
δT : −2.3% : δF : −5.69%
Scenario: DD
World
δT : −18.1% : δF : −7.65%
Afrotropical
δT : −3.7% : δF : −5.94%
Australian
δT : −4.2% : δF : −9.66%
Nearctic
δT : −1.4% : δF : −4.42%
Neotropical
δT : −6.7% : δF : −5.75%
Indo−Malay
δT : −4.9% : δF : −11.73
Palearctic
δT : −2.3% : δF : −5.69%
PC1 (66.64%)
PC2 (13.24%)
Quantile change
30%
0
−30%

C) Reptiles

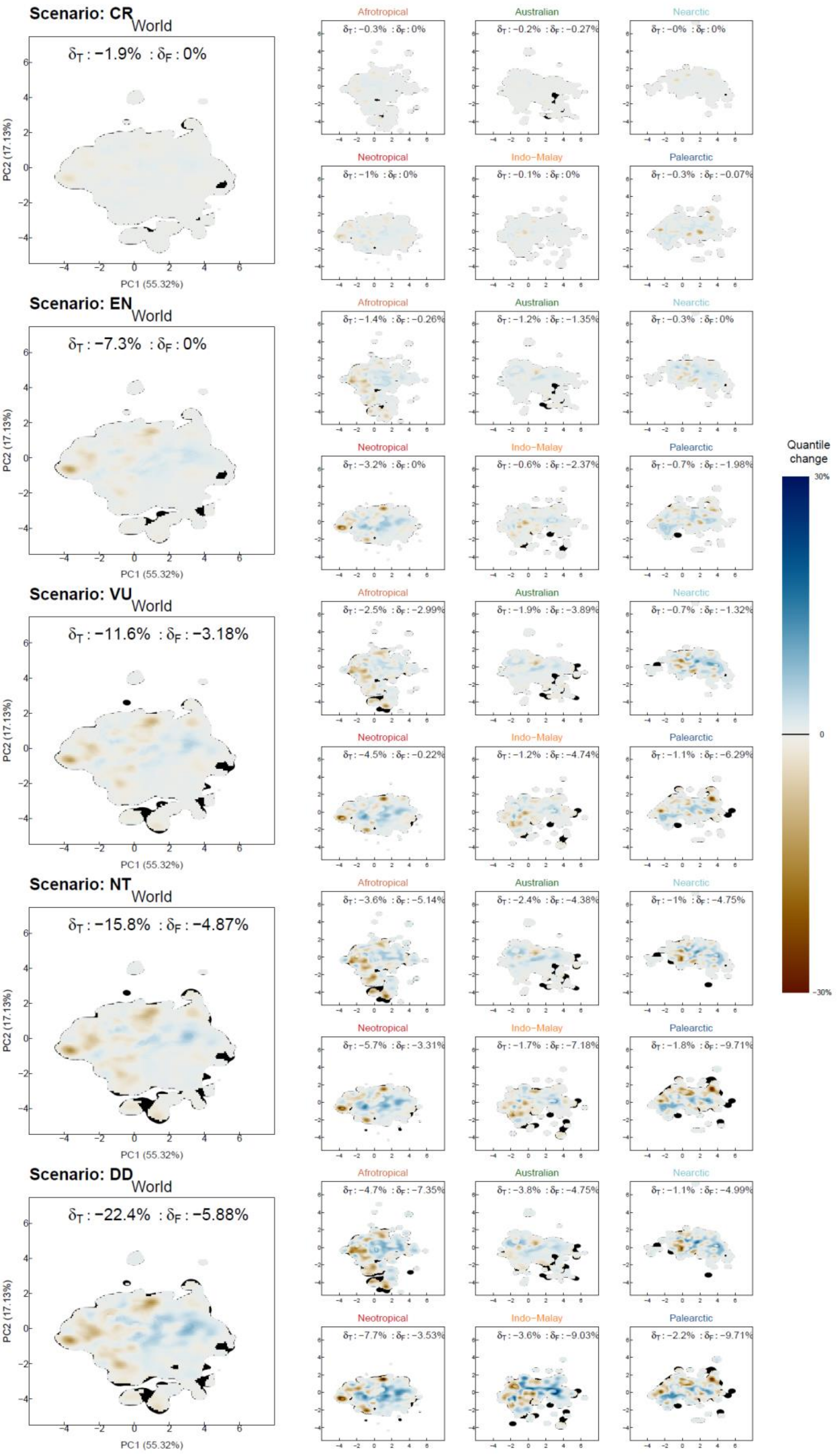

Scenario: CR
World
δT : −1.9% : δF : 0%
Afrotropical
δT : −0.3% : δF : 0%
Australian
δT : −0.2% : δF : −0.27%
Nearctic
δT : −0% : δF : 0%
Neotropical
δT : −1% : δF : 0%
Indo−Malay
δT : −0.1% : δF : 0%
Palearctic
δT : −0.3% : δF : −0.07%
Scenario: EN
World
δT : −7.3% : δF : 0%
Afrotropical
δT : −1.4% : δF : −0.26%
Australian
δT : −1.2% : δF : −1.35%
Nearctic
δT : −0.3% : δF : 0%
Neotropical
δT : −3.2% : δF : 0%
Indo−Malay
δT : −0.6% : δF : −2.37%
Palearctic
δT : −0.7% : δF : −1.98%
Scenario: VU
World
δT : −11.6% : δF : −3.18%
Afrotropical
δT : −2.5% : δF : −2.99%
Australian
δT : −1.9% : δF : −3.89%
Nearctic
δT : −0.7% : δF : −1.32%
Neotropical
δT : −4.5% : δF : −0.22%
Indo−Malay
δT : −1.2% : δF : −4.74%
Palearctic
δT : −1.1% : δF : −6.29%
Scenario: NT
World
δT : −15.8% : δF : −4.87%
Afrotropical
δT : −3.6% : δF : −5.14%
Australian
δT : −2.4% : δF : −4.38%
Nearctic
δT : −1% : δF : −4.75%
Neotropical
δT : −5.7% : δF : −3.31%
Indo−Malay
δT : −1.7% : δF : −7.18%
Palearctic
δT : −1.8% : δF : −9.71%
Scenario: DD
World
δT : −22.4% : δF : −5.88%
Afrotropical
δT : −4.7% : δF : −7.35%
Australian
δT : −3.8% : δF : −4.75%
Nearctic
δT : −1.1% : δF : −4.99%
Neotropical
δT : −7.7% : δF : −3.53%
Indo−Malay
δT : −3.6% : δF : −9.03%
Palearctic
δT : −2.2% : δF : −9.71%
PC1 (55.32%)
PC2 (17.13%)
Quantile change
30%
0
−30%

D) Amphibians

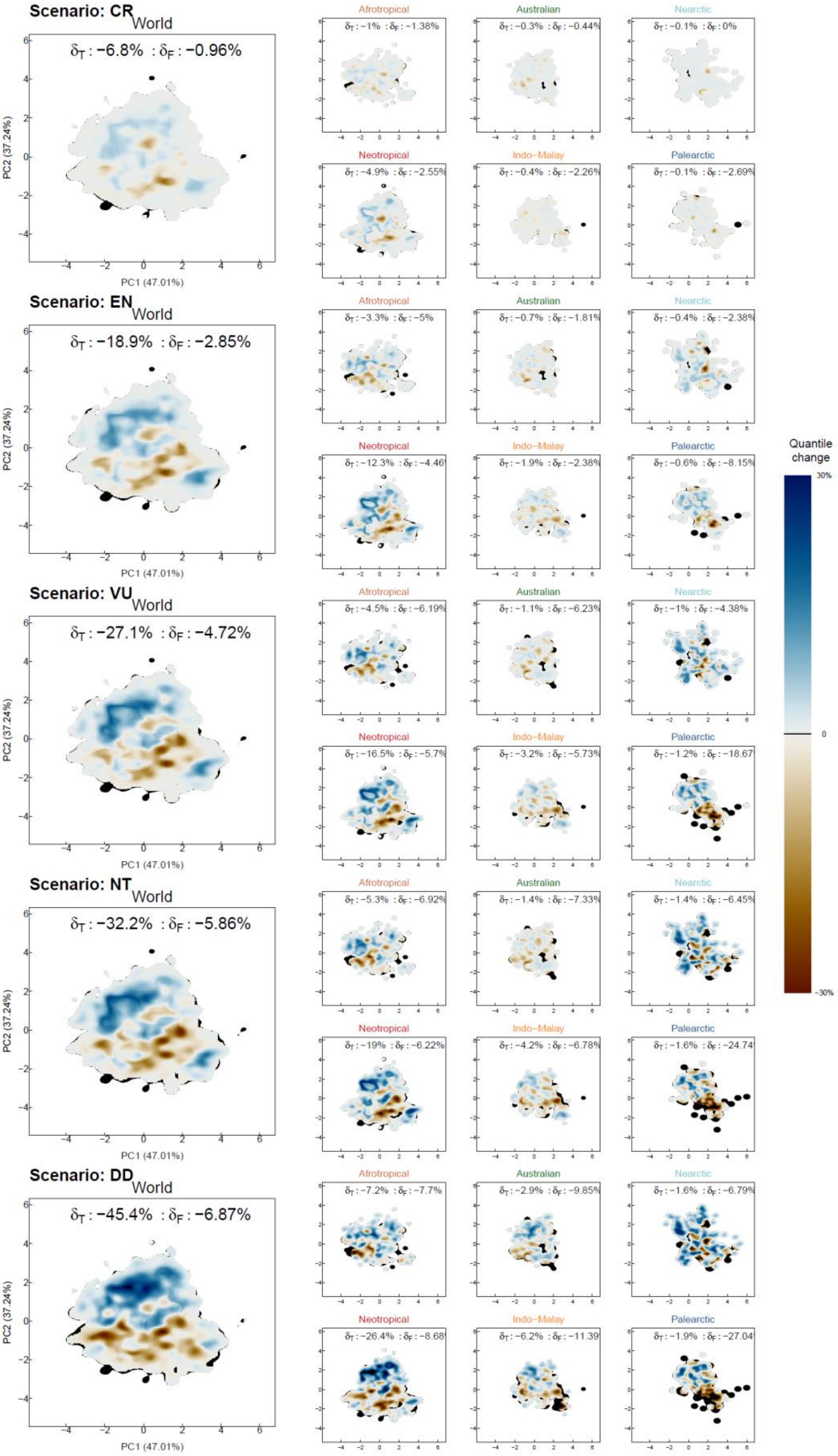

E) Freshwater fish

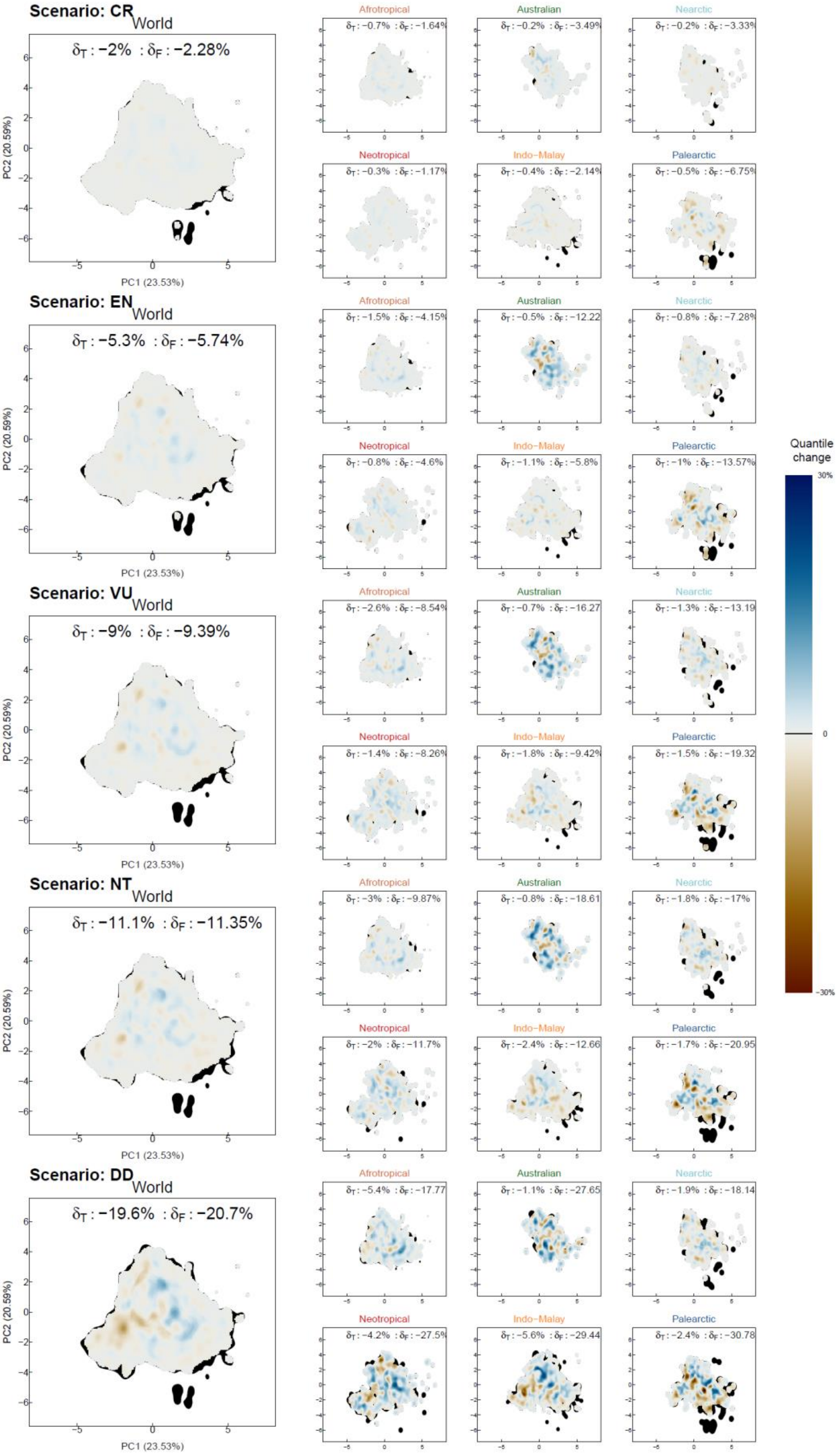

Supplementary Fig 6. Shifts in the functional diversity after the simulated extinction of threatened species for the six taxonomic groups. Each panel represents the functional spectra of a taxonomic group in a realm or in the world. For each taxonomic group (A-E), the shifts in functional diversity is shown for the six biogeographic realms and the world according to the scenario considered. Differences (expressed in quantiles changes) are calculated between the functional spectra of species assessed by IUCN Red List before and after removing species classified as threatened (see definition in the Methods). Brown tones reflect the threatened functional space after projected extinctions (i.e. areas representing trait values becoming relatively less frequent at the realm scale), and blue tones reflect the favourable functional space after extinctions (i.e. areas representing trait values becoming relatively more frequent at the realm scale). Black areas represent the lost functional space after extinctions. For each panel, the title shows the proportion of species (δT) and the proportion of total functional space (δF) that would be lost after extinction (expressed as a percentage of the current taxonomic and functional richness, respectively).

A) Without IUCN data.

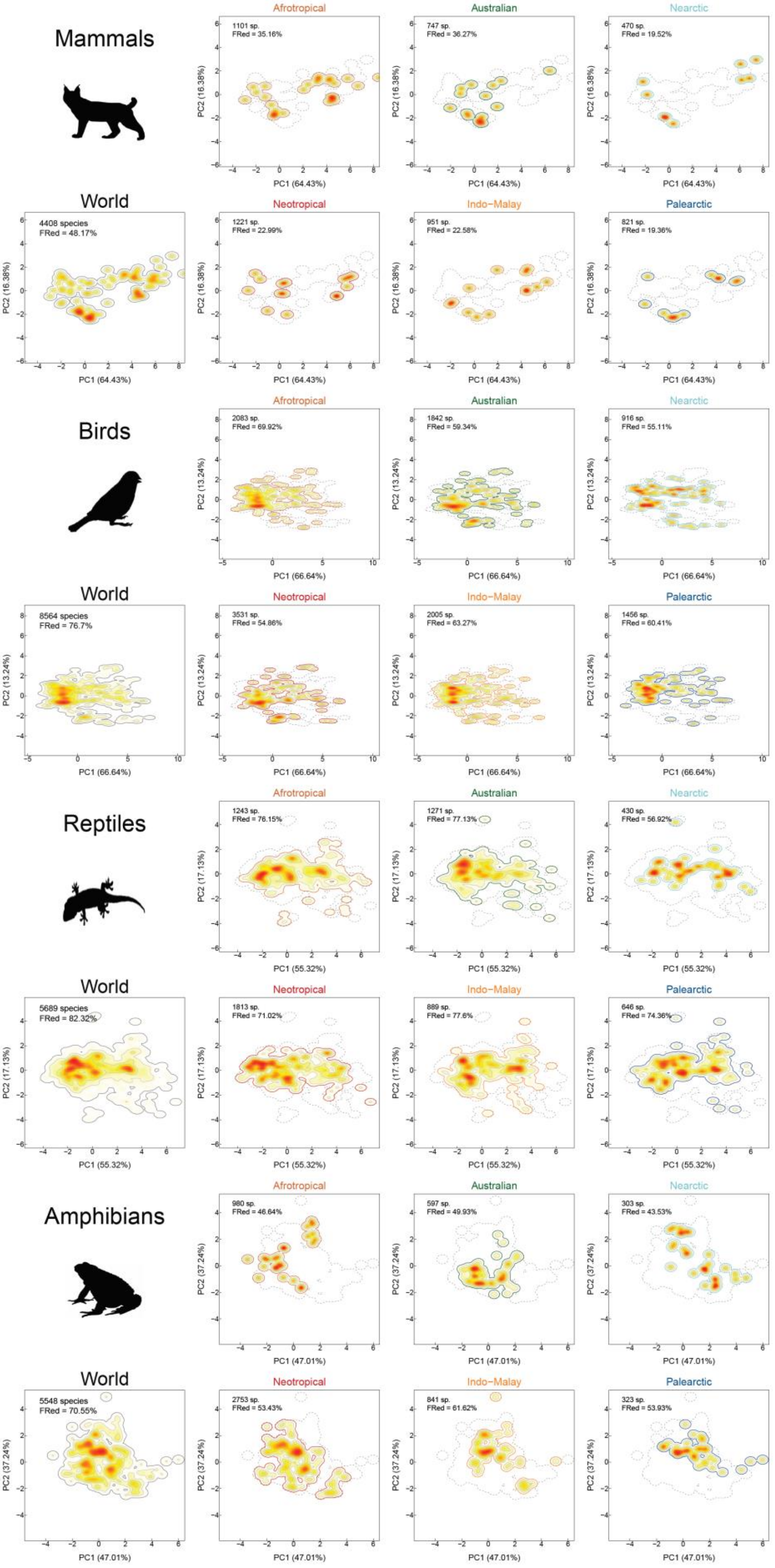

B) Without WWF (or Burgin et al. for mammals).

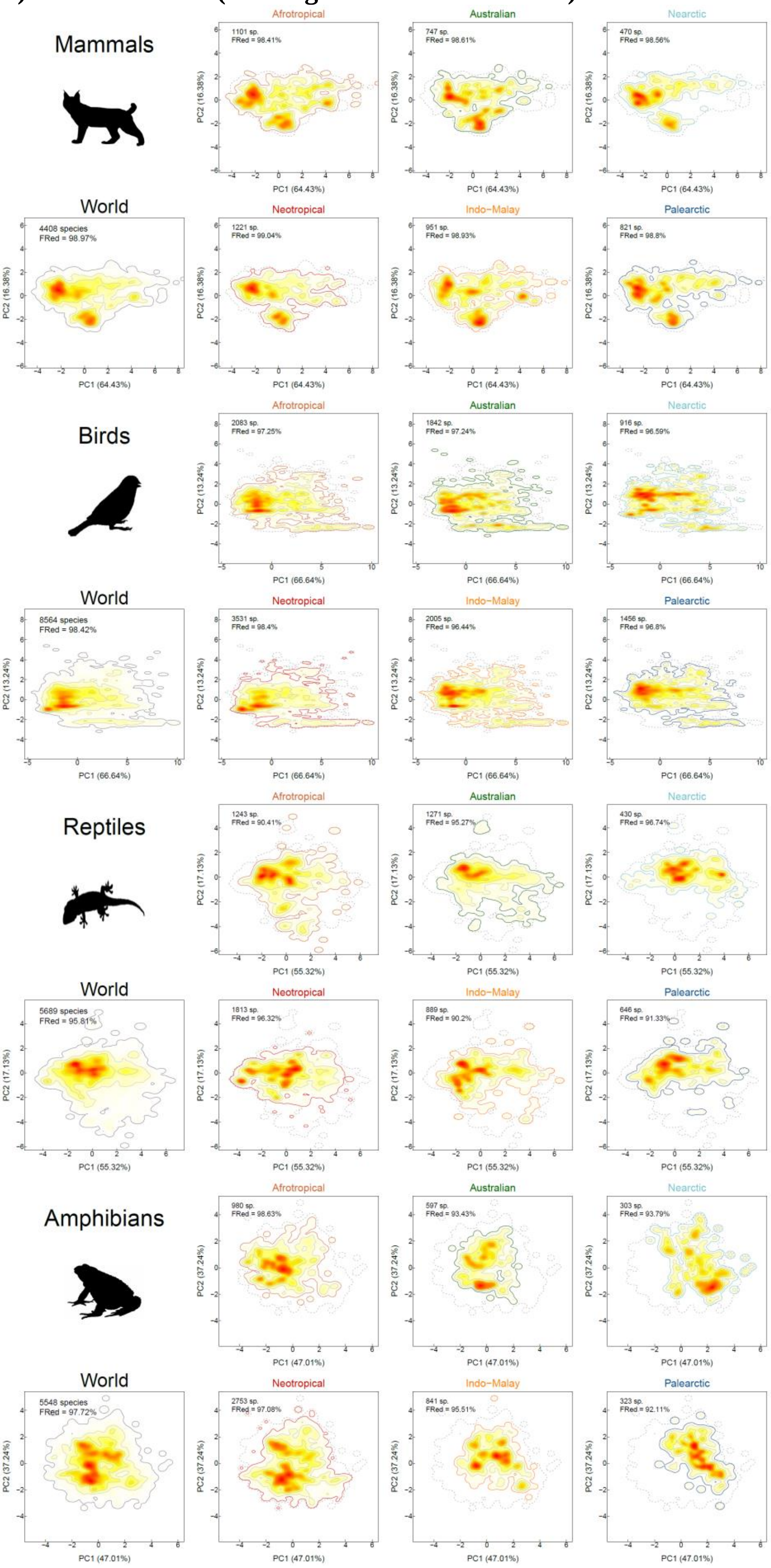

C) Without GBIF data.

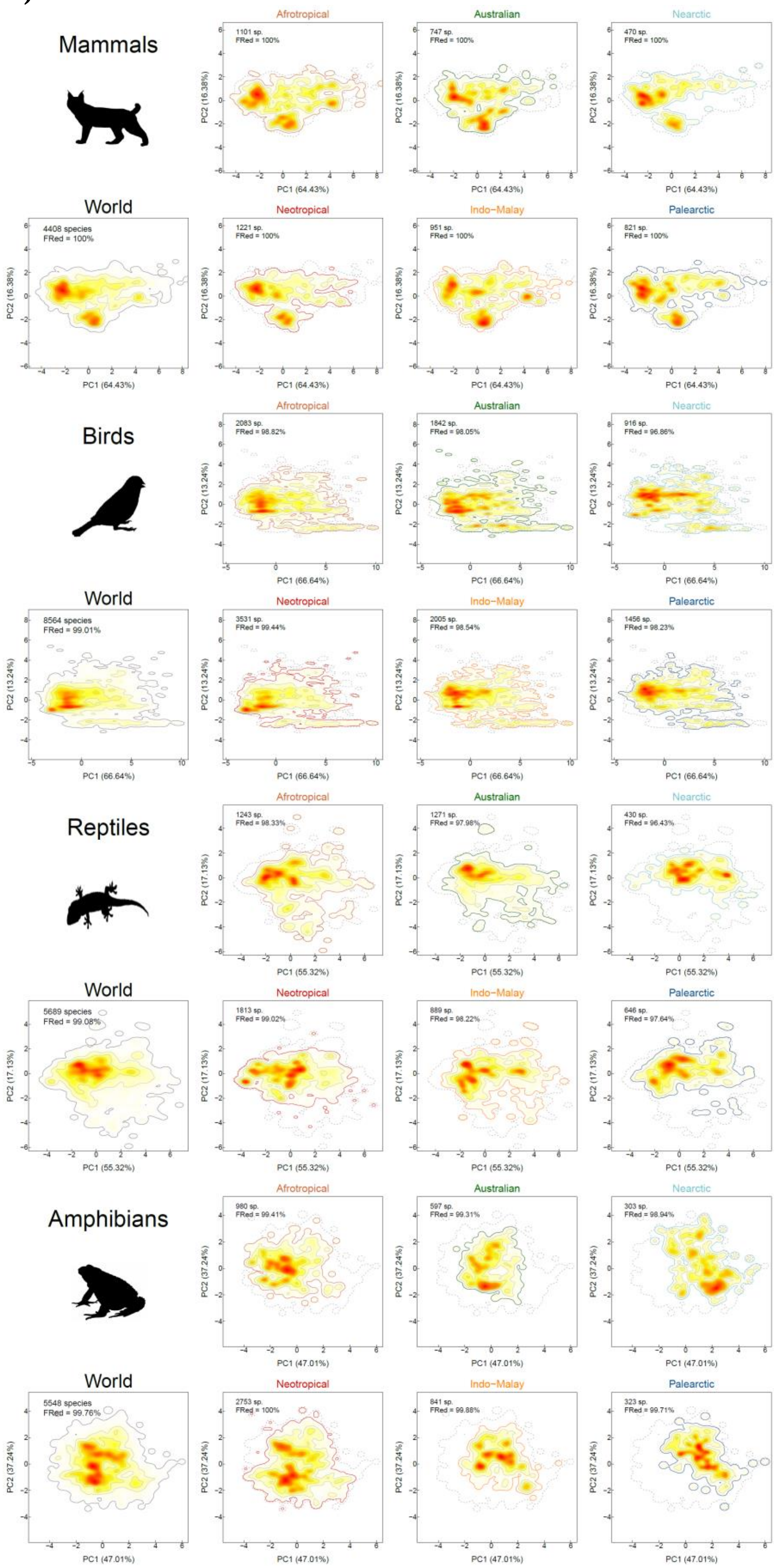

Supplementary Fig 7. Functional spectrum of four taxonomic groups in the six biogeographic realms and the world using a subset of species with spatial occurrences. Each panel represents the functional spectra of a taxonomic group in a realm or in the world. Probabilistic species distributions in the spaces are defined by the two first principal components of the PCA considering different functional traits for each group (see the Methods for details). A similar colour gradient is applied in Figure 1. For each taxonomic group in each realm, we compared the functional overlap between the functional spaces built with all species with spatial occurrences and the functional space built with all species except the species retrieved from the IUCN spatial data (A), or the species retrieved from WWF (B), or the species retrieved from GBIF (C). We measured the degree of functional redundancy (FRed) between the two subsets using the functional dissimilarity (FDiss). FRed corresponds to 1-FDiss and is expressed as a percentage. FRed is indicated in each panel for each taxonomic group. Silhouettes were downloaded from PhyloPic (www.phylopic.org). Figures have been made using R (codes are available online, see Code Availability).

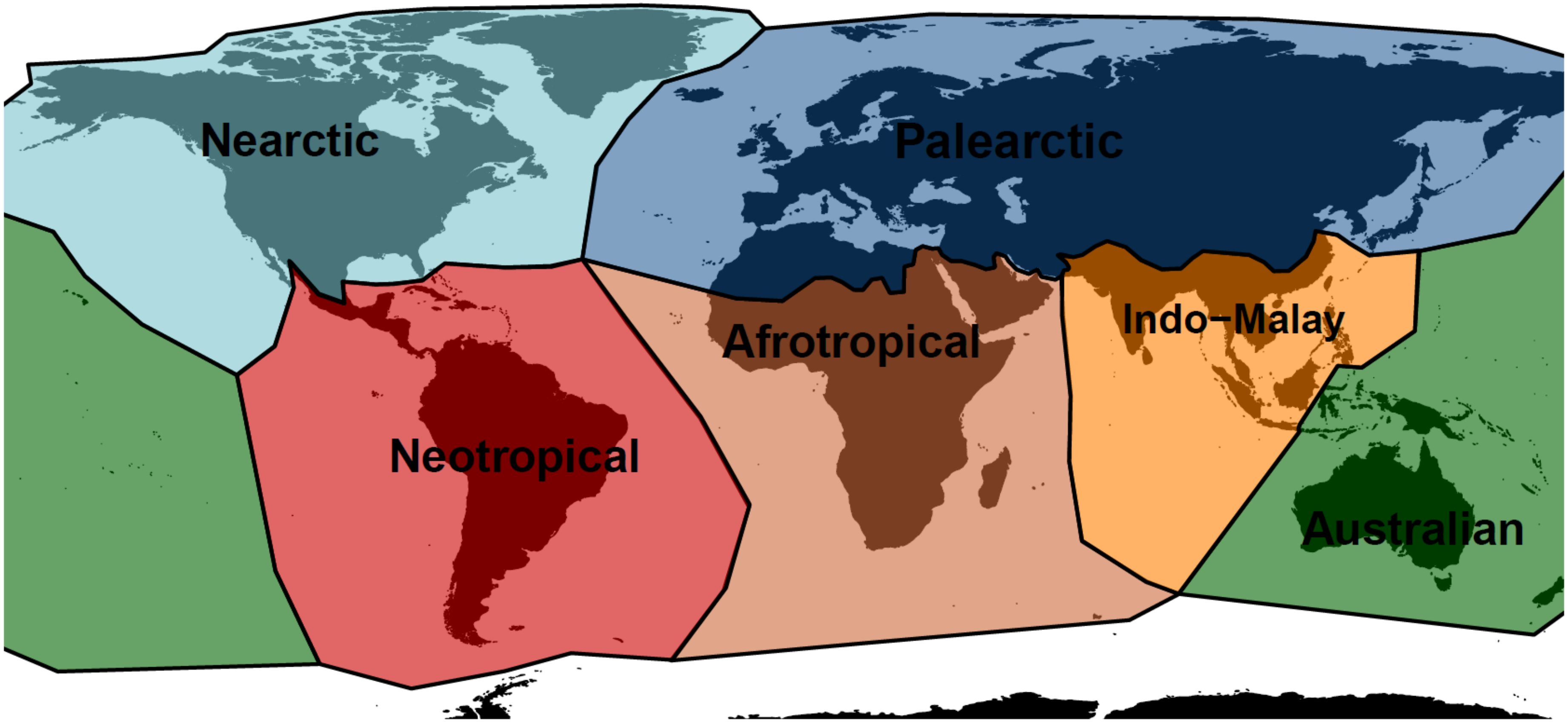

Supplementary Fig 8. Geographical limits of the six biogeographic realms. Geographical limits of the biogeographic realms adapted from ref.[3], freely available online (https://ecoregions2017.appspot.com). We combined Australasian and Oceania realms in one unique realm since the number of records was too low in Oceania to consider as an independent realm. We thus called it the Australian realm, following the terminology of ref.[4], avoiding any confusion. Figures have been made using R (codes are available online, see Code Availability).

A) Scenario CR

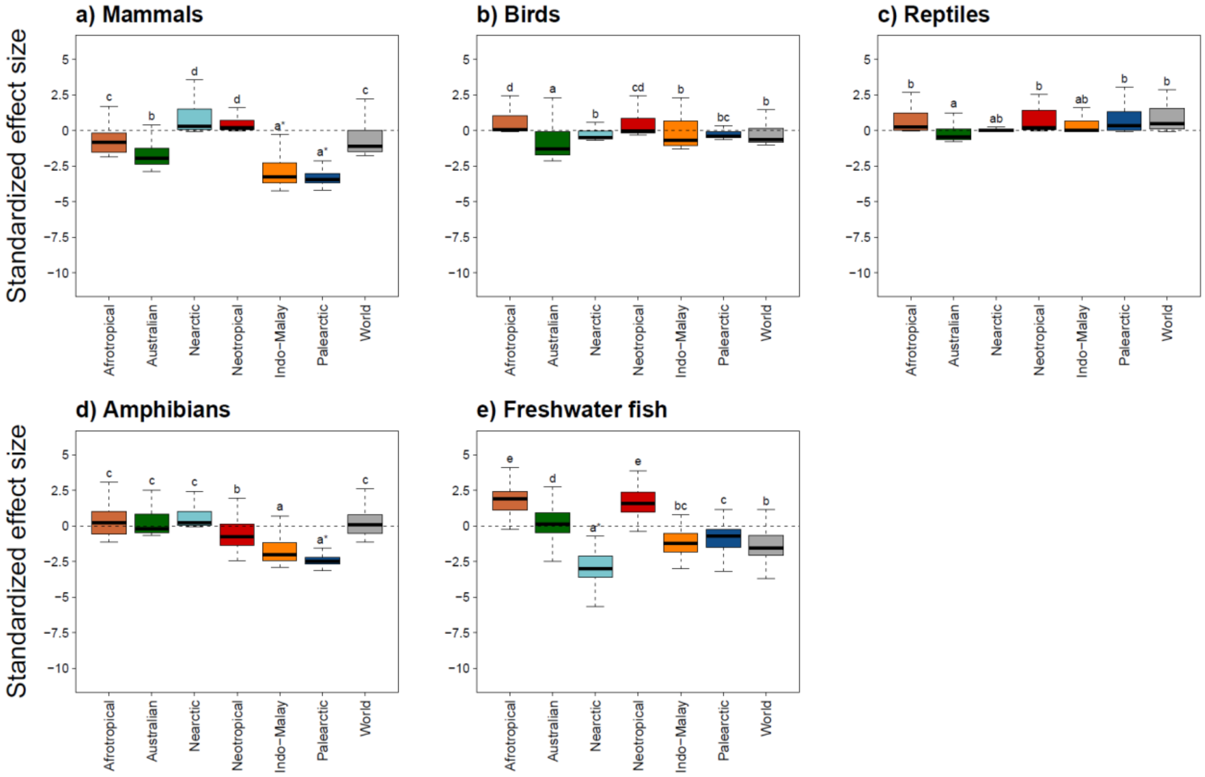

B) Scenario EN

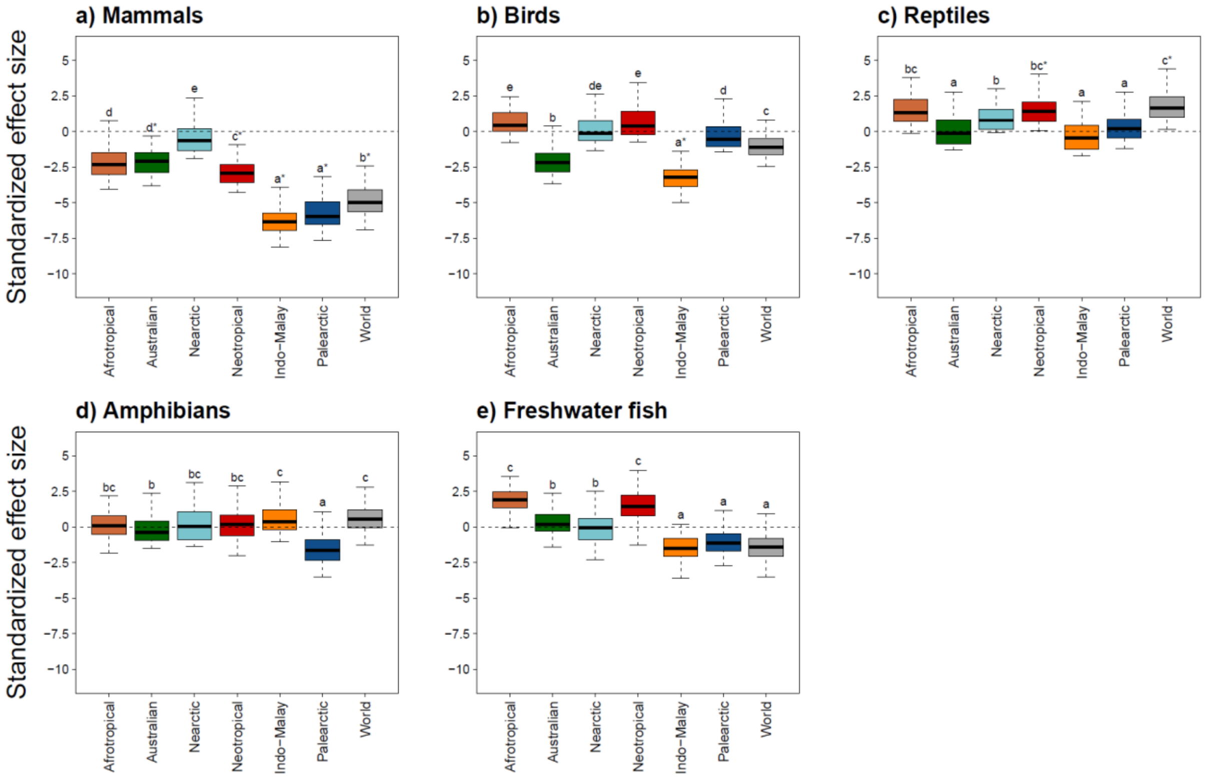

C) Scenario VU

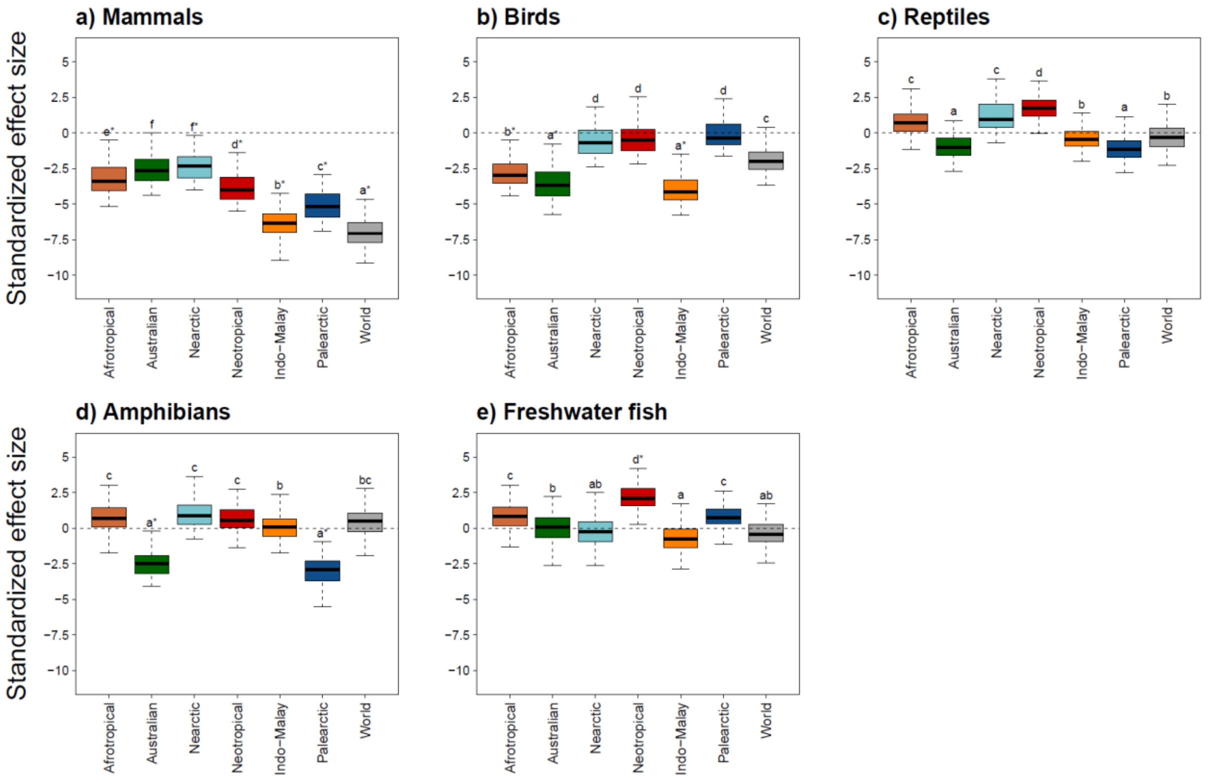

D) Scenario DD

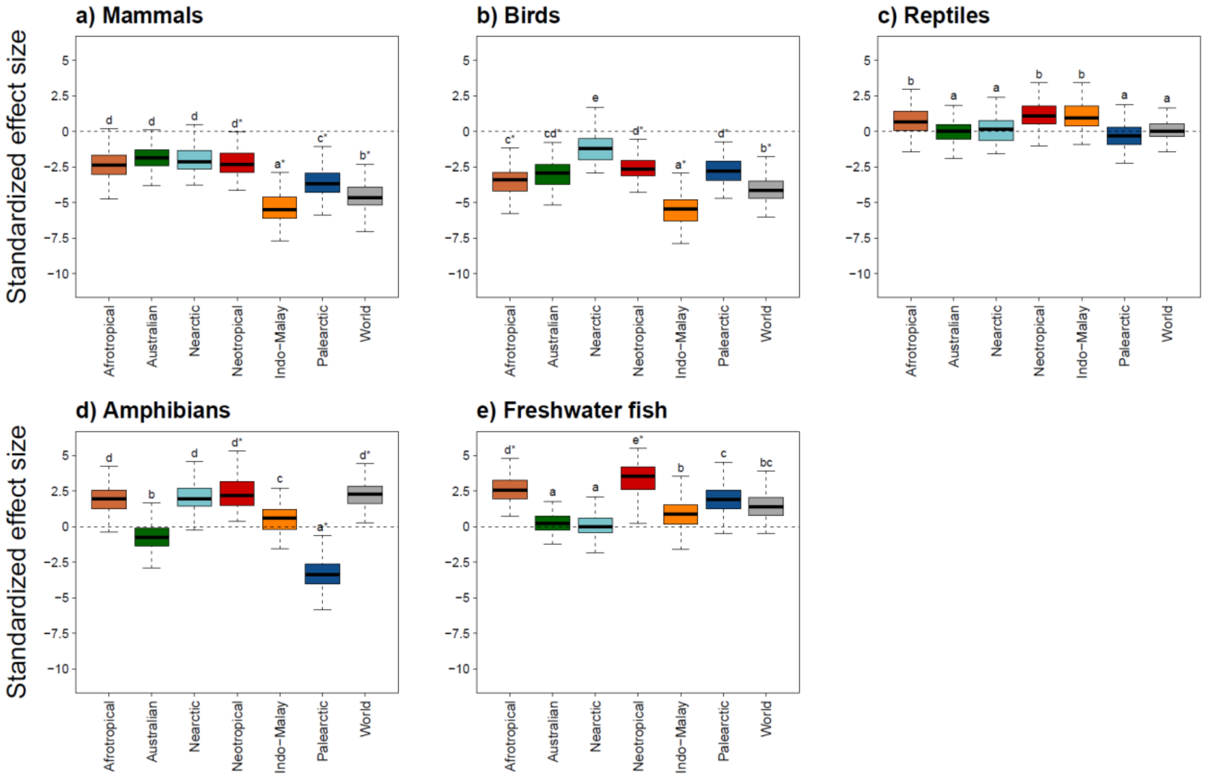

Supplementary Fig 9. Standardized effect size (SES) distribution for the vertebrates in the six biogeographic realms following the IUCN classes of threatened species. For each scenario (A-D) and taxonomic group (panels a-e), we tested whether the SES values of the potential loss in functional richness of each biogeographic realm were significantly different from each other using multiple

pairwise comparison tests. SES distributions were obtained using a bootstrapping procedure (n=1000 repetitions, see the Methods for details). We show for each group a compact letter displays of all pairwise comparisons with a significance-level at 5%. The SES significantly different than expected under null models is identified by an asterisk.

References

1. Carmona, C. P. *et al.* Erosion of global functional diversity across the tree of life. *Sci. Adv.* **7**, eabf2675 (2021).

2. Burgin, C. J., Colella, J. P., Kahn, P. L. & Upham, N. S. How many species of mammals are there? *J. Mammal.* **99**, 1–14 (2018).

3. Olson, D. M. *et al.* Terrestrial Ecoregions of the World: A New Map of Life on Earth. *Bioscience* **51**, 933 (2001).

4. Lévêque, C., Oberdorff, T., Paugy, D., Stiassny, M. L. J. J. & Tedesco, P. A. Global diversity of fish (Pisces) in freshwater. *Hydrobiologia* **595**, 545–567 (2008).
