# Supplementary material for: Extinction of threatened vertebrates will lead to idiosyncratic changes in functional diversity across the world
Source: Nat Commun. 2021 Aug 27;12:5162. doi: 10.1038/s41467-021-25293-0 (PMC8397725; doi:10.1038/s41467-021-25293-0)
Supplement: Supplementary file 2 — Description of Additional Supplementary Files [file 41467_2021_25293_MOESM2_ESM.pdf]

File name: Supplementary Data 1

Description: The proportion of species shared by each database for the six biogeographic realms and the world

File name: Supplementary Data 2

Description: Potential loss of functional diversity for the vertebrates according to scenarios of potential extinction of threatened species
