# Supplementary material for: Extinction of threatened vertebrates will lead to idiosyncratic changes in functional diversity across the world
Source: Nat Commun. 2021 Aug 27;12:5162. doi: 10.1038/s41467-021-25293-0 (PMC8397725; doi:10.1038/s41467-021-25293-0)
Supplement: Supplementary file 5 — Reporting Summary [file 41467_2021_25293_MOESM5_ESM.pdf]

nature research

Corresponding author(s): *Double-blind peer review submissions: write DBPR and your manuscript number here instead of author names.*

Last updated by author(s): Jun 4, 2021

Reporting Summary

Nature Research wishes to improve the reproducibility of the work that we publish. This form provides structure for consistency and transparency in reporting. For further information on Nature Research policies, see our Editorial Policies and the Editorial Policy Checklist.

Statistics

For all statistical analyses, confirm that the following items are present in the figure legend, table legend, main text, or Methods section.

n/a	Confirmed	
☐	☒	The exact sample size (*n*) for each experimental group/condition, given as a discrete number and unit of measurement
☐	☒	A statement on whether measurements were taken from distinct samples or whether the same sample was measured repeatedly
☐	☒	The statistical test(s) used AND whether they are one- or two-sided<br>*Only common tests should be described solely by name; describe more complex techniques in the Methods section.*
☐	☒	A description of all covariates tested
☐	☒	A description of any assumptions or corrections, such as tests of normality and adjustment for multiple comparisons
☐	☒	A full description of the statistical parameters including central tendency (e.g. means) or other basic estimates (e.g. regression coefficient) AND variation (e.g. standard deviation) or associated estimates of uncertainty (e.g. confidence intervals)
☐	☒	For null hypothesis testing, the test statistic (e.g. *F*, *t*, *r*) with confidence intervals, effect sizes, degrees of freedom and *P* value noted<br>*Give P values as exact values whenever suitable.*
☒	☐	For Bayesian analysis, information on the choice of priors and Markov chain Monte Carlo settings
☒	☐	For hierarchical and complex designs, identification of the appropriate level for tests and full reporting of outcomes
☐	☒	Estimates of effect sizes (e.g. Cohen's *d*, Pearson's *r*), indicating how they were calculated

Our web collection on statistics for biologists contains articles on many of the points above.

Software and code

Policy information about availability of computer code

Data collection	We used R packages: rredlist (v.0.5.5) for information about conservation status of species, V.PhyloMaker (v.0.1.0) for obtaining a phylogeny for vascular plants, and Taxonstand (v.2.1) and taxize (0.9.8) for taxonomic standardization of species names.
Data analysis	We used R version 3.6.0 (R Core Team 2019), along with the packages ade4 (v.1.7-13), ks (v.1.11.5), MissForest (v.1.4.), sp (and mgcv (v.1.8-29), phytools (v 0.6-60), TPD (v.1.1.0) for different calculations. The code used for analyses is available in Figshare as stated in the manuscript.

For manuscripts utilizing custom algorithms or software that are central to the research but not yet described in published literature, software must be made available to editors and reviewers. We strongly encourage code deposition in a community repository (e.g. GitHub). See the Nature Research guidelines for submitting code & software for further information.

Data

Policy information about availability of data

All manuscripts must include a data availability statement. This statement should provide the following information, where applicable:

- Accession codes, unique identifiers, or web links for publicly available datasets
- A list of figures that have associated raw data
- A description of any restrictions on data availability

Data and R scripts are available here. https://figshare.com/s/f076a046963c6f782f8d

Field-specific reporting

Please select the one below that is the best fit for your research. If you are not sure, read the appropriate sections before making your selection.

- [ ] Life sciences
- [ ] Behavioural & social sciences
- [x] Ecological, evolutionary & environmental sciences

For a reference copy of the document with all sections, see nature.com/documents/nr-reporting-summary-flat.pdf

Ecological, evolutionary & environmental sciences study design

All studies must disclose on these points even when the disclosure is negative.

Study description	We collected functional trait information from published and publicly available databases for five taxonomic groups: mammals, birds, reptiles, amphibians and functional fishes. For each group we performed a principal component analysis (PCA) based on the traits and defined its dimensionality (number of component providing non-redundant information). We also collected information on the conservation status of species from IUCN. We collected the occurrences of species in the six biogeographic realms from published and publicly available datasets for the five taxonomic groups. We further performed simulations of extinctions and compared the patterns of occupation of the PCA space before and after extinctions for the six biogeographic realms, as well as with null models in which threatened species are randomly selected from the global pool of species.
Research sample	rait information for 4,953 species of mammals, 9,802 species of birds and 6,567 species of reptiles was obtained from the Amniote database (Myhrvold et al. 2015). We used the AmphiBIO database (Oliveira et al 2017) to get data for 6,776 species of amphibians. Morphological traits for 10,705 species of strictly freshwater fishes were obtained from Toussaint et al. (2016), whereas fish body length and body mass were taken from FishBase (Froese and Pauly, accessed December 2019). We used the IUCN spatial data occurrence, WWF WildFinder (ver. Jan 06) to get spatial occurrence of birds, reptiles and amphibians (22,718 species), Burgin et al (2018) for mammals (4,718 species), and Tedesco et al. (2017) for freshwater fishes. For all these groups we obtained conservation status information from the IUCN red list.
Sampling strategy	We used all information available for all groups (described in research sampling).
Data collection	Data was collected from the aforementioned databases by Aurele Toussaint and Carlos P. Carmona
Timing and spatial scale	All data was collected during 2020. In the case of databases that are updated with some frequency (e.g. IUCN red list, GBIF), we collected data in December 2020 to ensure that data was as much up to date as possible.
Data exclusions	Some observations were exclude from the GBIF databases in the curation process. we filter out the records with clearly false locality coordinates (e.g. latitude equal longitude, both latitude and longitude equal 0, and longitude/latitude outside possible range). In addition, we removed the records from living specimens (i.e. from zoos, botanical gardens), conserved specimens (i.e. museums), and unknown sources.
Reproducibility	We did not perform any experiment. All our data is based on computer analyses. We provide the code to reproduce the results. Since there are some random processes included in the performed null model, results cannot be exactly reproduced, but main conclusions should be very stable.
Randomization	This is not relevant to our study since we do not perform any experiment.
Blinding	Blinding was not done since all data was collected from databases

Did the study involve field work? [ ] Yes [x] No

Reporting for specific materials, systems and methods

We require information from authors about some types of materials, experimental systems and methods used in many studies. Here, indicate whether each material, system or method listed is relevant to your study. If you are not sure if a list item applies to your research, read the appropriate section before selecting a response.

Materials & experimental systems

n/a	Involved in the study	
[x]	[ ]	Antibodies
[x]	[ ]	Eukaryotic cell lines
[x]	[ ]	Palaeontology and archaeology
[x]	[ ]	Animals and other organisms
[x]	[ ]	Human research participants
[x]	[ ]	Clinical data
[x]	[ ]	Dual use research of concern

Methods

n/a	Involved in the study	
[x]	[ ]	ChIP-seq
[x]	[ ]	Flow cytometry
[x]	[ ]	MRI-based neuroimaging
